# Enhanced Reaction Kinetics in Sodium‐Ion Batteries Achieved by 3D Heterostructure CoS_2_/CoS with Self‐Induced Internal Electric Field

**DOI:** 10.1002/advs.202502241

**Published:** 2025-04-25

**Authors:** Jin Liang, Jiawen Sun, Xin Cao, Xiaoshan Li, Xiaoyi Chen, Ruizhe Xing, Jie Kong

**Affiliations:** ^1^ MOE Key Lab of Materials Physics and Chemistry in Extraordinary Conditions Shaanxi Key Lab of Macromolecular Science and Technology School of Chemistry and Chemical Engineering Northwestern Polytechnical University Xi'an 710072 P. R. China; ^2^ Key laboratory of Flexible Electronics of Zhejiang Province Ningbo Institute of Northwestern Polytechnical University 218 Qingyi Road Ningbo 315103 P. R. China; ^3^ Research & Development Institute of Northwestern Polytechnical University in Shenzhen Sanhang Science &Technology Building No.45th, Gaoxin South 9th Road Nanshan Shenzhen 518063 P. R. China

**Keywords:** 3D honeycomb‐like construction, efficient mass transfer, heterostructure CoS2/CoS, internal electric field, Ultrafast sodium‐ion storage

## Abstract

The sluggish charging and restricted mass transfer of cobalt‐based sulfides have provoked in cycling stability, poor rate, and low initial coulombic efficiency, impeding their practical application. Developing electronic configurations and heterostructures are effective methods to improve conductivity and accelerate mass transfer. In this work, heterostructured carbon/cobalt sulfides embedded in honeycomb‐like nitrogen‐doped carbon (HC@CoS_2_/CoS/NC) were proposed as a cost‐effective strategy. These composites feature interconnected channels, facilitating rapid electron transport and efficient electrolyte diffusion. This self‐induced internal electric field design of HC@CoS₂/CoS/NC enhanced the charge movement, inherent conductivity and optimized the electrochemical kinetics as anode materials. Theoretical calculations indicate that the development of heterostructures with self‐induced internal electric fields is crucial for improving the charge particle/electron movement during the charge–discharge cycles of sodium‐ion batteries (SIBs), leading to enhanced Na^+^ diffusion. This anode demonstrated a high specific capacity of 809.0 mAh g^−1^ at 0.1 A g^−1^, retaining a capacity of 465.2 mAh g^−1^ after 700 cycles at 15 A g^−1^. When paired with Na_3_V_2_(PO_4_)_3_, the full‐cell maintained a specific capacity of 108.9 mAh g^−1^ after 200 cycles at 1.0 A g^−1^. This research presents an effective approach for developing transitional metal sulfide heterostructures as high‐performance anode materials for SIBs.

## Graphical abstract

A novel HC@CoS_2_/CoS/NC composite with optimized heterointerfaces is successfully synthesized as an ultrastable and fast‐charging anode material for SIBs. This composite features a 3D architecture, characterized by highly dispersed CoS_2_/CoS/NC nanoparticles embedded within honeycomb carbon nanosheets. Honeycomb carbon materials with extensive surfaces and oxygen‐containing functional groups have been utilized as both active substrates and conductive platforms, significantly enhancing the overall conductivity. Furthermore, HC@CoS_2_/CoS/NC offers numerous heterogeneous interfaces for Na^+^ migration and enhances electrochemical kinetics. The carefully designed hierarchical nanostructures provided stress buffering and facilitated ion transport, effectively extending the cycling lifespan. The HC@CoS_2_/CoS/NC composites demonstrated remarkable Na^+^ storage capability, high reversible capacity, and superior prolonged cycling ability.

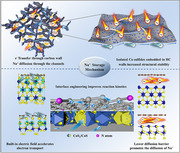

## 1 Introduction

Transition metal sulfides (TMSs) have emerged as highly promising anode materials for sodium‐ion batteries (SIBs). Among the anode materials for TMSs in SIBs, cobalt (Co)‐based materials, such as CoS_2_ (870 mAh g^−1^) and CoS (590 mAh g^−1^), are recognized as favorable options.^[^ ^1^ ^]^ These materials possess theoretical capacities that surpass those of commonly used hard carbon materials.^[^ ^2^, ^3^, ^4^ ^]^ Furthermore, the metal‐sulfur bonds in these materials are weaker and offer better conductivity than metal oxides, enhancing their reversibility and reaction kinetics.^[^ ^5^ ^]^ These merits have increased the research value of Co‐based sulfide materials in the realm of SIB anode materials.

Nonetheless, Co‐based sulfide materials encounter their inherently poor electrical conductivities and sluggish ionic transport kinetics. These limitations lead to suboptimal battery cycle and rate performances, significantly impeding their application, particularly in fast‐charging scenarios driven by increasing market demand.^[^ ^6^, ^7^ ^]^ Additionally, achieving excellent ionic and electronic conductivities is essential for fast‐charging electrodes.^[^ ^8^ ^]^ Various strategies have been reported to address the drawbacks of cobalt sulfide materials and to develop high‐power batteries, including the construction of nanostructured electronic configurations^[^ ^9^, ^10^ ^]^ and heterojunction strategies.^[^ ^11^, ^12^ ^]^ Fan et al. employed cobalt sulfide in combination with VS_4_ nanodots to fabricate a heterogeneous structured electrode material (CoS_2_/NC@VS_4_), which displayed desirable cycle stability as an anode for SIBs.^[^ ^13^ ^]^ Feng et al. assembled MXene nanosheets into hollow spheres on which CoS_2_ nanoparticles embedded in N‐doped carbon were grown (MXene@CoS_2_/NC), and the hollow structure buffered the volume expansion of the CoS_2_.^[^ ^14^ ^]^ Practically, the combination of these methods has a synergistic effect in promoting comprehensive electrochemical performance (e.g., specific capacity, cycling stability, and rate capability).^[^ ^15^ ^]^ Specifically, a combination of nanostructure design and carbon matrix material hybridization commonly results in improved electrochemical characteristics. Carbon materials can act not only as conductive additives to elevate electronic conductivity but also as stress buffering matrices to ameliorate serious volume changes in the anode during cycling.^[^ ^16^, ^17^, ^18^ ^]^

Heterostructure construction significantly enhances the reaction kinetics because a local built‐in electric field is spontaneously produced at the heterointerface, where the electronic properties are markedly improved, leading to a low ion diffusion barrier to boost the ion‐electron migration rate.^[^ ^19^ ^]^ Moreover, charge reassignment appearing at the heterointerfaces can increase the number of active sites for additional reversible capacity.^[^ ^20^ ^]^ For example, Min et al. synthesized a dual‐transition metal CoS_2_–SnO_2_@rGO heterostructure to accelerate the kinetic reaction and alleviate the volume change during charge/discharge, benefitting from the small grain size, high specific surface area effects, and heterointerfaces for the ion storage of CoS_2_–SnO_2_.^[^ ^21^ ^]^ Xu et al. synthesized a Ni‐doped FeSe_2_/Fe_3_Se_4_ heterojunction porous structure (NF_11_S/C) via selenization of a Fe‐based MOF precursor doped with Ni.^[^ ^22^ ^]^ More active sites for Na^+^ storage, supplied by N‐doped carbon, furnished a higher electronic state at the Fermi level, thereby increasing the specific capacity.^[^ ^23^ ^]^ Therefore, it is feasible to construct heterostructures to enhance the electrochemical properties of electrode materials.

In this study, a novel hierarchical porous cobalt sulfide carbon composite anode (HC@CoS_2_/CoS/NC) was obtained through a pyrolysis/sulfurization method. HC@CoS_2_/CoS/NC exhibits a 3D honeycomb‐like structure composed of confined carbon nanosheets and cobalt sulfide nanoparticles. This 3D honeycomb‐like framework not only facilitates rapid electron transport through the honeycomb walls but also provides interconnected channels for efficient electrolyte diffusion into the interior electrodes. The graphene‐like 2D honeycomb walls effectively protected the cobalt sulfide nanoparticles from degradation and accommodated volume variation during charge‐discharge cycles. Additionally, the pore surfaces were enriched with oxygen‐containing functional groups from both the HC and N‐doped carbon phases, offering extra storage sites for Na^+^. The built‐in electric field effect in the heterostructures of CoS_2_/CoS was demonstrated through theoretical calculations, which enhanced charge transfer and increased ionic transfer kinetics. Consequently, HC@CoS_2_/CoS/NC exhibited remarkable ultrafast Na^+^ storage, including a high reversible capacity (809.0 mAh g^−1^ at 0.1 A g^−1^), excellent rate capability (430.5 mAh g^−1^ at 20 A g^−1^), and exceptional long‐term cycling stability (531.4 mAh g^−1^ after 700 cycles at 10 A g^−1^). Furthermore, when coupled with a Na_3_V_2_(PO_4_)_3_ cathode, the full battery retained a specific capacity of 108.9 mAh g^−1^ after 200 cycles at 1.0 A g^−1^, providing valuable insight into the fabrication of heterostructured SIB anode materials.

## 2 Results and Discussion

### 2.1 Preparation and Characterizations

**Figure 1:**
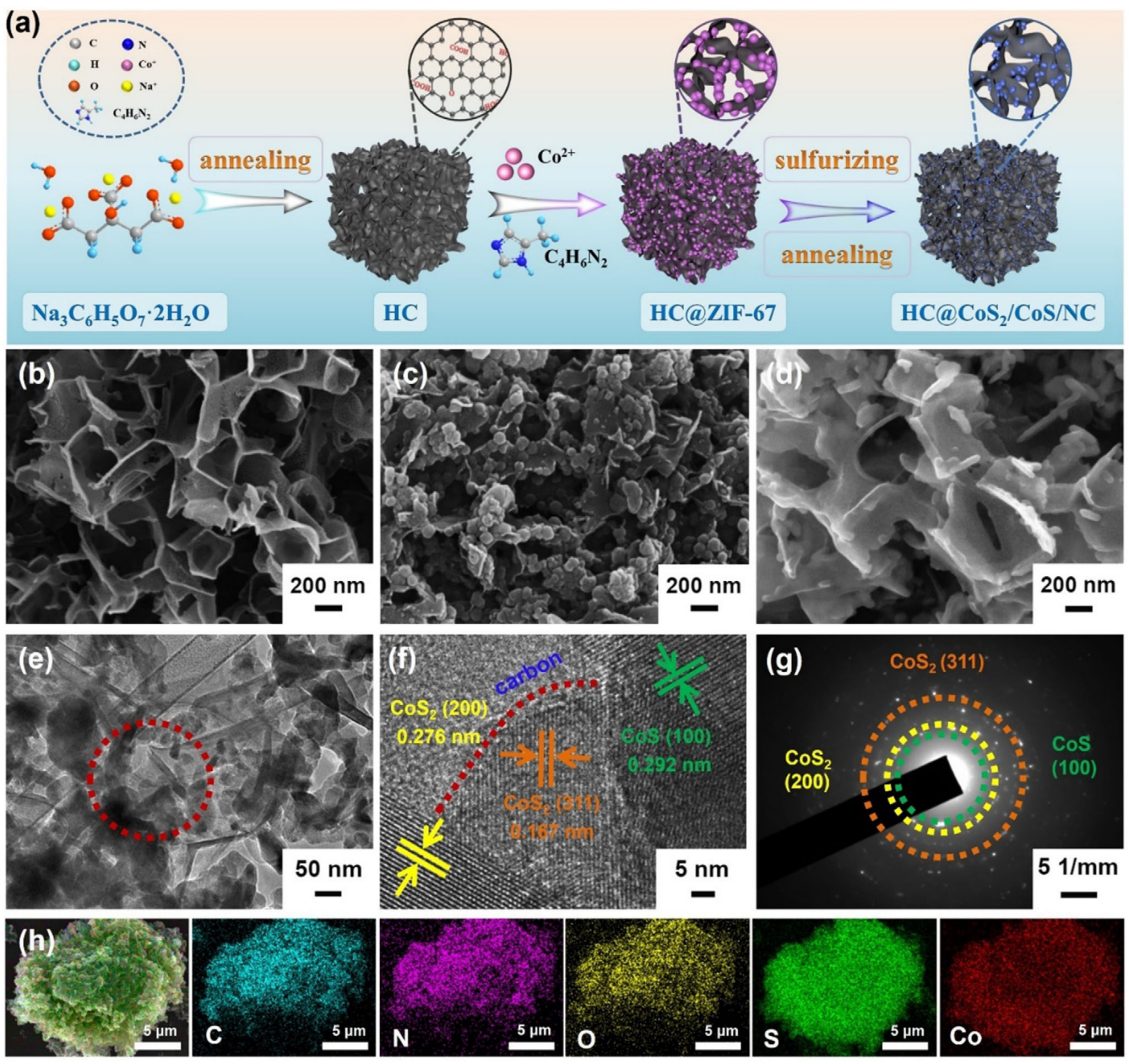
a) Schematic diagram of the preparation process of HC@CoS_2_/CoS/NC; SEM images of b) HC and c) HC@ZIF‐67; d) SEM, e) TEM, f) HRTEM, g) SAED, and h) elemental mapping images of HC@CoS_2_/CoS/NC.

**Figure** **1**a illustrates the synthesis strategy for HC@CoS_2_/CoS/NC composites. In a typical synthesis process, HC was initially prepared as a hard template for ZIF‐67 growth through calcination using sodium citrate, as described in a previous report.^[^ ^24^ ^]^ In this process, the organic ligands in the sodium citrate molecules undergo carbonization at specific calcination temperatures in an inert atmosphere. Subsequently, many carbon atoms gradually form carbon nanosheets by self‐assembly owing to the Na‐containing species acting as catalysts. And multilayer nanosheets with porous structures are cross‐linked to produce a continuous conductive honeycomb‐like network.^[^ ^25^, ^26^ ^]^ Subsequently, a co‐precipitation method was applied to deposit the MOFs onto the HC scaffold to produce HC@ZIF‐67 composites. Co^2+^ ions are anchored onto the surface of HCs through electrostatic interactions with superficial oxygen‐containing functional groups, which can further serve as nucleation sites.^[^ ^14^ ^]^ The HC@ZIF‐67 composites subsequently operate in situ on the surface of carbon after the addition of 2‐MIM, based on a simple chemical coordination reaction.^[^ ^27^ ^]^ Finally, HC@CoS_2_/CoS/NC was obtained through a carbonization/sulfurization procedure in the presence of sulfur powder to achieve component conversion under an argon atmosphere.

The obtained HC had a typical honeycomb‐like structure, as revealed by the SEM and TEM images (Figure 1b; Figure S1a–e, Supporting Information). Importantly, the honeycomb‐like structure endows the composites with the features of potent supporting ability and is suitable.^[^ ^28^ ^]^ Additionally, the selected‐area electron diffraction (SAED) image in Figure S1f (Supporting Information) indicates the disordered nature of the carbon matrix. Figure S1e (Supporting Information) shows a regional short‐range ordered pattern, benefitting from the increased electrical conductivity compared with disordered amorphous carbon.^[^ ^25^ ^]^ Figure S2a–c (Supporting Information) confirms that HC consists of carbon and oxygen, and Fourier transform infrared spectroscopy (FT‐IR) characterization revealed the existence of oxygen‐containing groups (Figure S2d, Supporting Information). In addition to functioning as redox sites for Na^+^ capture, oxygen groups play a critical role in improving the wettability of negative materials to electrolytes, thereby enhancing the rate capability.^[^ ^29^, ^30^ ^]^ ZIF‐67 was homogeneously anchored in the HCs to generate HC@ZIF‐67 intermediates, the characteristics of which are shown in Figure 1c and Figures S3 and S4 (Supporting Information). The intermediates were subsequently transformed into HC@CoS_2_/CoS/NC via thermal decomposition and sulfuration treatment. Figure 1d,e images showed that the CoS_2_/CoS/NC particles were anchored to the surface of the HC. The HC@CoS_2_/CoS/NC composites generally possess a 3D honeycomb architecture consisting of carbon nanosheets and confined CoS_2_/CoS nanoparticles. This 3D HC not only offers adequate honeycomb walls for fast electron transport but also supplies 3D hexagonal channels for Na^+^ diffusion. In addition, 2D honeycomb walls effectively protect cobalt sulfide nanoparticles from deterioration and accommodate volume variation during charge/discharge cycles, enabling ultrahigh rate capabilities and ultralong cycle stabilities.^[^ ^31^, ^32^ ^]^

**Figure 2:**
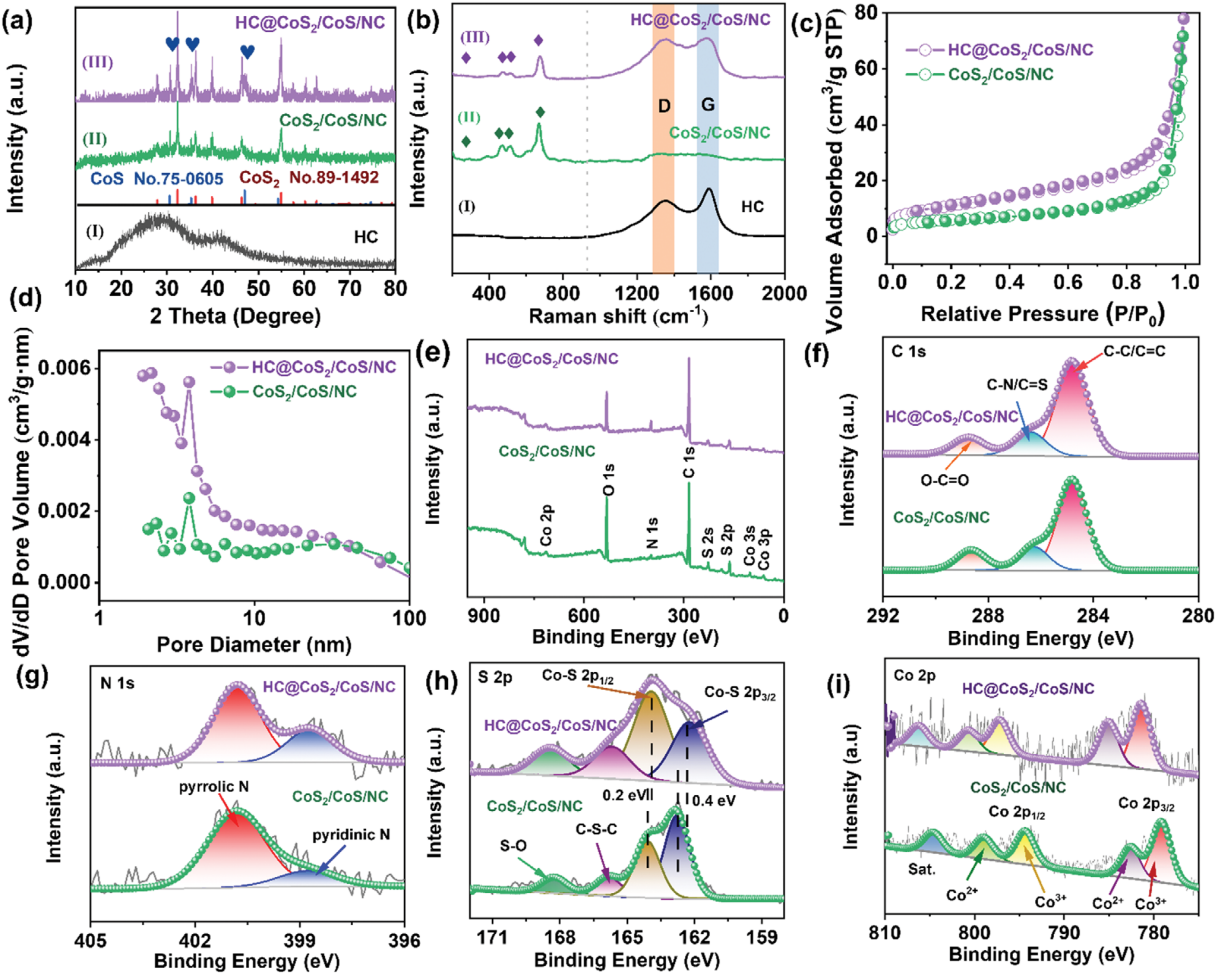
a) XRD patterns and b) Raman spectra of three samples: HC@CoS_2_/CoS/NC and CoS_2_/CoS/NC; c) nitrogen adsorption/desorption isotherms; d) pore size distributions; XPS spectra: e) full scan, f) C 1s, g) N 1s, h) S 2p, and i) Co 2p.

In the high‐resolution TEM (HRTEM) image of the HC@CoS_2_/CoS/NC composites (Figure 1f), clear lattice fringes of 0.276, 0.167, and 0.292 nm were observed, which were consistent with the (200) and (311) flatness of CoS_2_ and the (100) plane of CoS.^[^ ^33^ ^]^ Clear interfaces between CoS_2_ and carbon, CoS and carbon, and CoS_2_ and CoS were detected and marked by red lines, confirming the formation of heterostructures of CoS_2_/C, CoS/C, and CoS_2_/CoS. This heterogeneous interface facilitates the electrochemical performance of Na^+^ storage.^[^ ^34^ ^]^ The elemental mapping of HC@CoS_2_/CoS/NC in Figure 1h indicated even distributions of C, N, O, S, and Co. For comparison, CoS_2_/CoS/NC was synthesized, and its characterization is shown in Figure S5a,b (Supporting Information). Furthermore, Figure S5c,d (Supporting Information) showed distinct interplanar distances of 0.273 and 0.292 nm, which correspond to the (200) facet of CoS_2_ and (100) plane of CoS. The crystal structures and compositions of the three samples were characterized using X‐Ray Diffraction (XRD) (**Figure** **2**a). Figure 2a I reveals that the HC had a typical soft carbon texture accompanied by a humpy hill at ≈24.6–26.2° and a weak peak at 43°, which was related to the (100) diffraction of partially disordered graphene stacks.^[^ ^35^ ^]^ The curves of both CoS_2_/CoS/NC (Figure 2a II) and HC@CoS_2_/CoS/NC (Figure 2a III) matched well with the standard card of CoS_2_ (JCPDS No. 89–1492).^[^ ^14^ ^]^ Notably, both composites demonstrated diffraction characteristics at 30.5°, 35.3°, and 47.0°, which are in accordance with the (100), (101), and (102) planes of CoS (JCPDS No. 75–0605), indicating the existence of CoS.^[^ ^34^ ^]^ The peak intensity associated with CoS_2_ implies that CoS_2_ becomes dominant in HC@CoS_2_/CoS/NC and CoS_2_/CoS/NC.^[^ ^36^ ^]^ The presence of carbon in these composites was further confirmed using Raman spectroscopy (Figure 2b). The peak at 1351 cm^−1^ was assigned to the D band, which refers to a locally disordered and defective structure, notably at the margins of the graphene and graphite flakes, whereas the peak at 1581 cm^−1^ was located in the G band (graphitic structure) of carbon.^[^ ^6^ ^]^ Figure 2b I showed the soft carbon characteristics, in which the intense D and G bands were attributed to the deficiencies and nanosized graphene crystallites.^[^ ^37^ ^]^ The *I_D_/I_G_* values of HC@/CoS_2_/CoS/NC and HC were 2.21 and 2.12, respectively. However, the D and G bands for CoS_2_/CoS/NC, compared to those of the other two composites, are obscure. Generally, a higher relative value of *I_D_/I_G_* suggests a low graphitization level and a high percentage of structural defects, which indicates that HC@/CoS_2_/CoS/NC possesses many more storage sites for ion storage.^[^ ^38^ ^]^ In addition, the peaks at 291, 471, and 670 cm^−1^ were characteristic of CoS_2_,^[^ ^39^ ^]^ and very similar peaks were observed at 471, 513, and 668 cm^−1^, which were in agreement with the CoS phase.^[^ ^40^, ^41^ ^]^

The N_2_ adsorption–desorption isotherms of HC, HC@CoS_2_/CoS/NC, and CoS_2_/CoS/NC, displayed in Figure 2c,d and Figure S6 (Supporting Information), were categorized as H3 type with a noticeable hysteresis loop, suggesting the occurrence of micro/mesoporous structures.^[^ ^42^ ^]^ The Brunauer–Emmett–Teller–specific surface areas were 262.8, 39.9, and 21.9 m^2^ g^−1^ for HC, HC@CoS_2_/CoS/NC, and CoS_2_/CoS/NC, respectively (Table S1, Supporting Information). The specific surface area of HC@CoS_2_/NC is approximately twice that of CoS_2_/CoS/NC, which was enhanced by the introduction of HC, thereby improving electrolyte permeability and providing more active sites for the reaction. Additionally, an appropriate pore size can improve the efficiency of Na^+^ intercalation.^[^ ^43^ ^]^ The above characterizations confirmed the well‐established hierarchical structure of HC@CoS_2_/CoS/NC. The honeycomb‐like carbon layer enhances the electrical conductivity of cobalt sulfide nanoparticles, mitigates physical stress during cycling, and reduces the solubility of the active species generated during electrochemical reactions. The optimized 3D conductive carbon framework with multiple channels facilitated efficient ion and electron transport. C, N, O, S, and Co were measured in both HC@CoS_2_/CoS/NC and CoS_2_/CoS/NC (Figure 2e). The C 1s spectrum (Figure 2f) contained three major peaks at 284.8, 286.4, and 288.7 eV, which were attributed to the C═C/C─C, C─N/C═S, and O─C═O bonds, respectively.^[^ ^44^, ^45^ ^]^ In the N 1s spectrum (Figure 2g), two fitting peaks at 398.7 and 400.8 eV belonged to pyridinic and pyrrolic N, respectively, and both kinds of N were considered active sites improving the electrochemical capacity.^[^ ^46^ ^]^ In the S 2p spectrum (Figure 2h), the peaks at 162.2 and 163.9 eV for HC@CoS_2_/CoS/NC were attributed to the S 2p_3/2_ and S 2p_1/2_ of the Co‐S band. The bonding energies of HC@CoS_2_/CoS/NC, compared with those of CoS_2_/CoS/NC, were negatively shifted (0.2 and 0.4 eV), indicating depressed Co─S bond energy as a result of the introduction of electronegative HCs to form heterostructures, increasing the conversion reaction kinetics.^[^ ^14^ ^]^ The characteristic peak at ≈168.4 eV corresponds to the S─O bond, which was mainly related to strong interactions with oxygen and water.^[^ ^47^ ^]^ The peak at 165.7 eV is assigned to C‐S‐C, which forms when cobalt sulfides/carbon compounds are annealed at high temperatures. This bonding helps to immobilize sulfur species, mitigating the volume expansion and aggregation issues of cobalt sulfide particles.^[^ ^48^, ^49^ ^]^ In the Co 2p spectrum (Figure 2i), the peaks at 780.3 and 795.8 eV were attributed to Co^3+^, resulting from minor surface oxidation. The peaks at 783.8 and 799.8 eV correspond to the Co^2+^ state.^[^ ^50^ ^]^ These obvious shifts were likely due to the formation of interfaces, which causes a redistribution of opposite charges.^[^ ^34^ ^]^

### 2.2 Sodium‐Ion (Na+) Storage Performance

**Figure 3:**
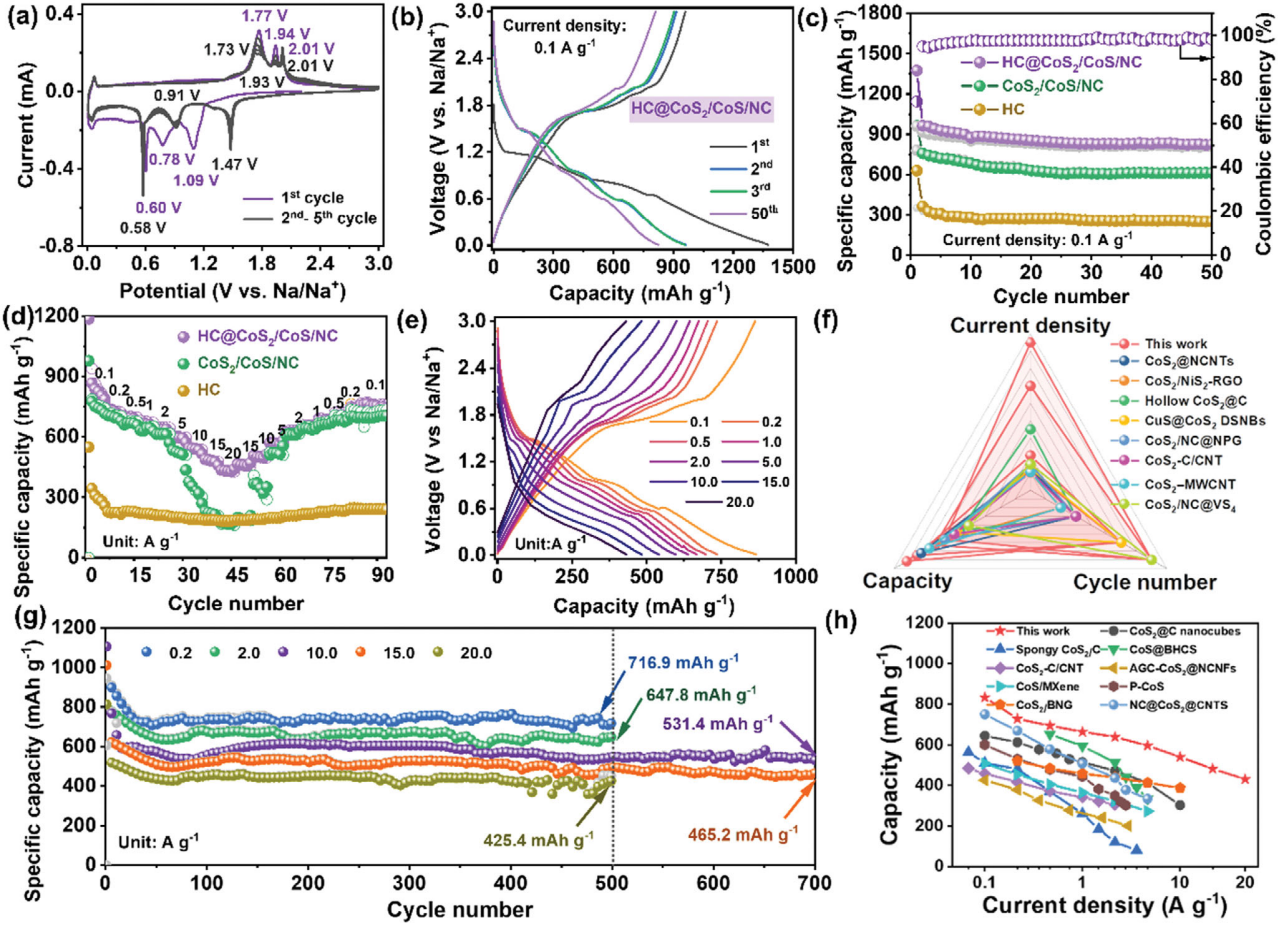
a) CV profiles at a scan rate of 0.1 mV s^−1^, b) charge‐discharge curves at 0.1 A g^−1^ in different cycles, c) cycling capabilities, d) rate performance of three samples and e) charge‐discharge curves at different current densities of HC@CoS_2_/CoS/NC; f) comparison of cycle performance with relative studies; g) long‐term cycling properties at different current densities of HC@CoS_2_/CoS/NC; h) comparison of rate performance with the reported CoSx‐based electrode.

The as‐prepared composites were assembled into CR2016‐type button half‐cells to evaluate their electrochemical properties. **Figure** **3**a shows the first five cyclic voltammetry (CV) plots of the HC@CoS_2_/CoS_2_/NC composites from 0.01 to 3 V at 0.1 mV s^−1^. Distinct cathodic and anodic peaks were observed, indicating multiple electrochemical reactions. During the initial cathodic sweep, three reduction peaks (at 1.09, 0.78, and 0.60 V) were assigned to the multistep conversion reaction of CoS_2_ to generate metallic Co and Na_2_S and the formation of a solid–electrolyte interface (SEI) layer, respectively. The subsequent oxidation peaks found at 1.77 to 2.01 V in the first anodic scan corresponded to the decomposition of Na_2_S and the formation of CoS_2_.^[^ ^14^, ^51^ ^]^ In the following cathodic process, the peak positions clearly shifted to 1.47, 0.91, and 0.58 V, which may be ascribed to structural reorganization after the initial sodiation.^[^ ^52^ ^]^ Compared with HC@CoS_2_/CoS/NC, the CV curve of CoS_2_/CoS/NC (Figure S7a, Supporting Information) did not exhibit any new peaks or disappearances. The largely overlapping CV curves of HC@CoS_2_/CoS/NC confirm the reversible uptake and release of sodium after an initial “activation,” suggesting a relatively stable electrochemical process.^[^ ^53^ ^]^ The capacity voltage plots of the HC@CoS_2_/CoS/NC composites for the first, second, and fifth cycles at 0.1 A g^−1^ are shown in Figure 3b. These plots were aligned with the cyclic voltammetry curves (Figure 3b) and were similar to those of CoS_2_/CoS/NC (Figure S7a, Supporting Information). In the initial cycle, a discharge capacity of 1374.2 mAh g^−1^ and a charge capacity of 960.2 mAh g^−1^ yielded a coulombic efficiency (CE) of 69.9%, reflecting the generation of the SEI film and the slow dissolution of polysulfide intermediates. The discharge capacity of 1374.2 mAh g^−1^ exceeded the theoretical specific capacity of cobalt sulfides (CoS_2_, 870 and CoS, 590 mAh g^−1^). This capacity, above the theoretical value, may be attributed to the additional Na storage capacity provided by redox reactions between oxygen‐containing functional groups and Na^+^.^[^ ^29^ ^]^ The CV and charge‐discharge curves of HC are displayed in Figure S8a (Supporting Information). Moreover, charge reassignment at heterointerfaces increases the number of active sites for additional reversible capacity.^[^ ^20^ ^]^ During the subsequent cycles, the capacity voltage plots (Figure 3b) largely overlapped, indicating that HC@CoS_2_/CoS/NC exhibited excellent stability.Figure 3c shows the cyclic stabilities of the three anodes at 0.1 A g^−1^. After the initial capacity decline, the HC@CoS_2_/CoS/NC anode demonstrated a reversible capacity of 915.2 mAh g^−1^ during the second charging process, maintaining a reversible capacity of 809.0 mAh g^−1^ after 50 cycles with a capacity retention of 88.4%. In comparison, CoS_2_/CoS/NC (Figure S7b, Supporting Information) and HC (Figure S8b, Supporting Information) exhibited capacity retentions of 81.7% and 76.1% with reversible charge capacity of 620.3 and 250.6 mAh g^−1^ after 50 cycles, respectively. These results demonstrated that HC@CoS_2_/CoS/NC possessed superior cycling stability. Furthermore, the HC@CoS_2_/CoS/NC anode exhibited excellent rate performance with average reversible capacities of 833.5, 728.2, 694.4, 664.0, 638.5, 596.2, 539.5, 481.7, and 430.5 mAh g ^−1^ at current densities of 0.1, 0.2, 0.5, 1, 2, 5, 10, 15, and 20 A g^−1^, respectively (Figure 3d). The charge‐discharge patterns of HC@CoS_2_/CoS/NC are illustrated in Figure 3e. Particularly, at higher densities of 5, 10, 15, and 20 A g^−1^, the capacities of CoS_2_/CoS/NC and HC were lower than those of HC@CoS_2_/CoS/NC, possibly because of the instability of the structure at higher densities (Figure S9b,c, Supporting Information).

The advantages of HC contributed to the excellent capability of HC@CoS_2_/CoS/NC. Moreover, the heterointerfaces between cobalt sulfides and carbon accelerate the reaction kinetics through the built‐in field, resulting in a superior cycling performance compared to other reported CoS and CoS_2_‐based electrodes for SIBs (Figure 3f; Table S2, Supporting Information). The long‐term cycling ability of HC@CoS_2_/CoS/NC characterized at high current densities of 10, 15, and 20 A g^−1^ released high reversible capacities of 531.4, 465.2, and 425.4 mAh g^−1^ after 500, 700, and 700 cycles, respectively (Figure 3g). Besides, it maintained high reversible capacities of 716.9, 647.8, and 637.9 mAh g^−1^ at 0.2, 2.0, and 5 A g^−1^ after 500, 500, and 300 cycles, respectively (Figure S10, Supporting Information). Compared with previously reported state‐of‐the‐art Co‐based chalcogenide anode materials for SIBs, HC@CoS_2_/CoS/NC demonstrated superior cycling stability and higher specific capacity, particularly at high current densities (Figure 3h; Table S3, Supporting Information), indicating significant potential for fast‐charging technology and longevity. Furthermore, the honeycomb‐like structure remained largely intact even after 700 cycles at 15 A g^−1^ (Figure S11, Supporting Information).

### 2.3 Kinetic Analysis

**Figure 4:**
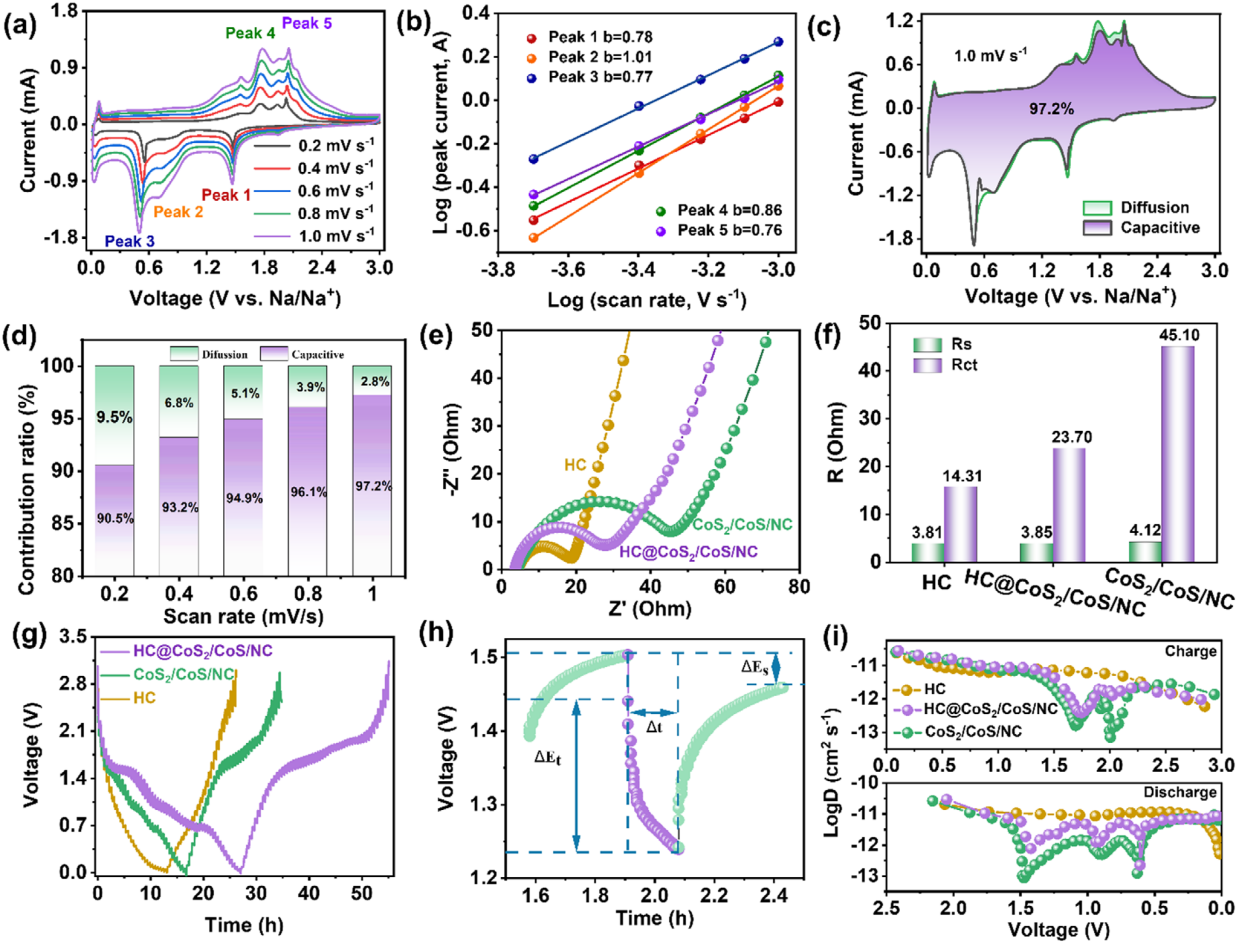
Kinetics analysis of the HC@CoS_2_/CoS/NC anode for SIB. a) CV files at a scan rate of 0.2–1.0 mV s^−1^, b) relationship and fitting plots of log(*i*) and log(*v*), c) capacitive contribution at 1.0 mV s^−1^ and d) capacitive and diffusion‐controlled contribution ratio at 0.2–1.0 mV s^−1^. e) nyquist plots and corresponding f) histograms of *R_s_* and *R_ct_* values, and g) GITT profiles of the three electrodes. h) enlarged local GITT curve for HC@CoS_2_/CoS/NC in a single current pulse: i) Na^+^ diffusion coefficient during the discharge–charge process for the three electrodes.

CV curves were recorded at scan rates ranging from 0.2 to 1.0 mV s^−1^ (**Figure** **4**a) to investigate the storage dynamics of Na^+^. The shape of the CV curves remained consistent, with the redox peak intensities increasing as the scan rate increased, suggesting a rapid response to fast charge transfer. It is widely accepted that materials with high surface areas and complex structures function as extrinsic pseudocapacitors.^[^ ^54^ ^]^ The redox capacitive contribution of HC@CoS_2_/CoS/NC was determined using the following equation:

(1) $$i=av^{b}$$

(2) $$\log\left(i\right)=b\log\left(v\right)+\log\left(a\right)$$

(3) $$i=k_{1}v+k_{2}v^{1/2}$$

where *v* is the scan rate (V s^−1^) and *i* (A) denotes the current associated with *v*. Equation (1) defines the relationship between *i* and *v* with constants a and b. The value of b ranges from 0.5 (diffusion‐controlled mechanism) to 1.0 (capacitive‐controlled process) and is determined from the slope of the log (*i*)‐log (*v*) plot using Equation (2) (Figure 4b). The b values for all five samples exceeded 0.75, indicating that the process was predominantly governed by the capacitance within the mixed kinetic regime. This suggests rapid Na^+^ intercalation/extraction and excellent cycling stability.^[^ ^55^ ^]^ Further quantification of the contribution rates was performed using Equation (3), where *k_1_v* and *k_2_v^1/2^* express the pseudocapacitive and ionic diffusion contributions, respectively.^[^ ^56^ ^]^ The pseudocapacitive contributions are 90.5%, 93.2%, 94.9%, 96.1%, and 97.2% at scan rates of 0.2, 0.4, 0.6, 0.8, and 1.0 mV s^−1^, respectively (Figure 4d). As the scan rate increased, the diffusion contribution decreased, while the contribution of the pseudocapacitive contributions increased. The exceptional 3D conductive network and numerous active sites of the HC@CoS_2_/CoS/NC composites resulted in high capacitive performance, enabling them to handle high‐density currents and thus exhibit superior rate performance. Figure 4c displayed the ratio of pseudocapacitive to ionic diffusion contributions at 1.0 mV s^−1^, indicating that pseudocapacitive charge storage predominantly controls the overall capacity at high scan rates, facilitating ultrafast Na^+^ transport kinetics during the desodiation/sodiation process.

The charge transfer kinetics of the HC@CoS_2_/CoS/NC electrodes were further investigated by electrochemical impedance spectroscopy (EIS) over a frequency range of 10^−2^ to 10^5^ Hz. Nyquist plots were fitted to extract parameters, including internal resistance (*R_s_*), charge transfer resistance (*R_ct_*), and Warburg impedance (*W_σ_*). *Rs* is the intercept of the semicircle with the real axis, *R_ct_* is the diameter of the semicircle in the high‐frequency region, and *W_σ_* is the slope of the inclined line in the low‐frequency region. According to previous studies, *R_s_* is associated with the resistance of the electrode connections, current collector, separator, and electrolyte. *R_ct_* represents the resistance at the electrode–electrolyte interface, whereas *W_σ_* is related to the diffusion of sodium ions.^[^ ^57^ ^]^ HC exhibited the lowest *R_ct_* value of 14.3 Ω, as illustrated in Figure 4e,f. Importantly, the HC@CoS_2_/CoS/NC electrode showed a significantly lower *R_ct_* of 23.7 Ω compared to the CoS_2_/CoS/NC electrode (45.1 Ω) before cycling, illustrating the successful construction of heterostructures with improved electric fields due to the inclusion of HC. Furthermore, the heterointerfaces formed can effectively reduce the charge‐transfer resistance, enhance the charge‐transfer kinetics, and boost the overall conductivity of the electrode.^[^ ^58^ ^]^ This effect was also observed after 50 and 100 cycles (Figure S12, Supporting Information).

The galvanostatic intermittent titration technique (GITT) was used to monitor the electrochemical reaction kinetics by measuring the Na^+^ diffusion coefficient (*D_Na+_*) during the sodiation/desodiation process. *D_Na+_* is calculated using the following equation (Equation 4):

(4) $$D_{Na^{+}}=\frac{4}{\pi \tau }{\left(\frac{m_{B}V_{m}}{M_{B}A}\right)}^{2}{\left(\frac{\Delta E_{s}}{\Delta E_{\tau }}\right)}^{2}$$

where *τ* (h) denotes the time during which the current pulse is applied; *ΔEs* (V) indicates the change in the steady‐state potential following the current pulse; *ΔEτ* (V) represents the variation in cell voltage during a constant current pulse in a single‐step GITT test (Figure 4h); m_B_ (g), M_B_ (g mol^−1^), and V_m_ (L mol^−1^) correspond to the mass, molecular weight, and molar volume of the active materials, respectively; and A (cm^2^) is the contact area between the electrolyte and electrode materials.^[^ ^59^ ^]^ Figure 4g indicated a complete GITT experiment for three electrodes during a single discharge/charge cycle, whereas the local GITT curve for a single current pulse was larger (Figure 4h). The HC sample exhibits the highest Na^+^ diffusion coefficient (10^−10.6^ to 10^−12.2^ cm^2^ s^−1^) and the lowest interfacial charge transfer resistance compared to other samples, as depicted in Figure 4i. Moreover, the *D_Na+_* of the HC@CoS_2_/CoS/NC electrode (10^−10.5^ to 10^−12.6^ cm^2^ s^−1^) exceeded that of the CoS_2_/CoS/NC electrode (10^−10.6^ to 10^−13.1^ cm^2^ s^−1^) during the sodiation/desodiation processes, revealing faster Na^+^ diffusion kinetics. This enhancement was attributed to the synergistic effects of the heterointerface, built‐in electric field porous surface, and 3D honeycomb‐like structure.^[^ ^1^ ^]^

### 2.4 Theoretical Calculation and Analyses

**Figure 5:**
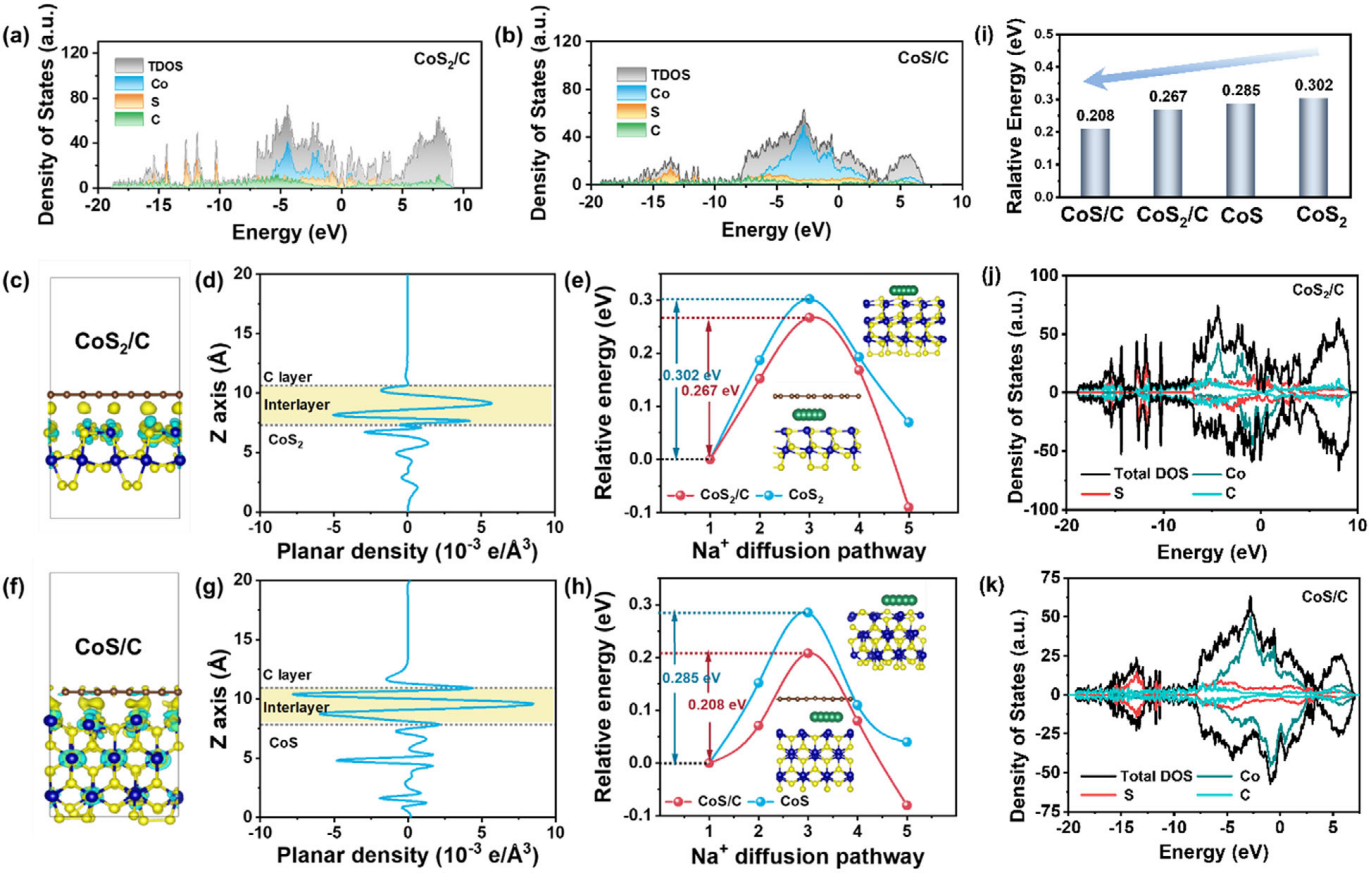
Calculated DOS of a) CoS_2_/C and b) CoS/C; CoS_2_ (200)/C: c) charge density difference, d) planar average charge density difference in the Z direction, and e) Na^+^ diffusion energy barrier profile; CoS (100)/C: f) charge density difference, g) planar average charge density difference in the Z direction, and h) Na^+^ diffusion energy barrier profile; i) energy barriers of the four samples; projected density of states for j) CoS_2_/C and k) CoS/C. Brown, blue, yellow, and green spheres represent C, Co, S, and Na, respectively.

To gain further insight into the improved electrochemical properties, the effects of heterostructures between cobalt sulfides and carbon were investigated using DFT calculations. Electronic conductivity is crucial for promoting fast electron transfer pathways during the redox processes. Initially, the density of states (DOS) was calculated to evaluate the merits of heterostructures between cobalt sulfides and carbon (**Figure** **5**a,b,j,k; Figure S13, Supporting Information). The DOS for both sulfides/carbon (CoS_2_/C and CoS/C) and pure cobalt sulfides (CoS_2_ and CoS) exhibit a non‐zero density of states at the Fermi level, confirming their metallic nature with continuous electronic states at this energy level.^[^ ^14^ ^]^ CoS_2_ exhibits metallic conductivity, characterized by a continuous overlap between its conduction and valence bands in the electronic structure. In contrast, CoS is typically a narrow‐bandgap semiconductor, featuring a well‐defined conduction band minimum and valence band maximum. Figure 5j,k shows that the CoS_2_/C composite exhibited a higher DOS than that of CoS_2_, with significant electron delocalization around the Fermi level, confirming the enhanced metallic conductivity.^[^ ^60^ ^]^ Similar results were observed for CoS/C compared to CoS, as shown in Figure 5b and Figure S13b (Supporting Information). The presence of heterostructures improves the overall conductivity, and accelerates electron transfer, and the dynamic phase of the conversion reaction, which contributes to the enhanced rate capability. In addition, the electron transfer behavior in the heterostructures was assessed using Work Functions. Variance analysis of the charge density (Figure 5c,f) revealed charge redistribution in the heterostructures between the cobalt sulfides and carbon layers. The influence of heterostructures on electron transfer due to the incorporation of a graphene‐like carbon layer was further demonstrated by the quantized distribution along the Z‐axis direction (Figure 5d,g). The observed peaks at the interlayer positions highlight the substantial electron transfer at the heterojunction surface, indicating significant charge redistribution.^[^ ^61^ ^]^

Another advantage of the interface between cobalt sulfides and the carbon layer is enhanced Na^+^ diffusion. When Na^+^ arrives at the anode material in batteries, it is primarily adsorbed onto the free surface of the crystal in the anode materials (here, the (200) plane of CoS_2_ and the (100) plane of CoS were used as the base) and subsequently undergoes multistep conversion reactions with the crystal.^[^ ^62^ ^]^ The diffusion paths and calculated results for cobalt sulfides (CoS_2_ and CoS) and cobalt sulfides/C (CoS_2_/C and CoS/C) are shown in Figure 5e,h. For the interfaces between carbon and cobalt sulfides, the energy barriers of CoS_2_/C and CoS/C were 0.267 and 0.208 eV, respectively, which were lower than those of pure CoS_2_ (0.302 eV) and CoS (0.285 eV) (Figure 5i). When CoS_2_ comes into contact with CoS, a built‐in electric field forms at the interface due to the difference in work functions. Resulting in band bending and the creation of a Schottky barrier. Electrons migrate from CoS to CoS_2_, forming a unidirectional carrier transport path. The formation of the Mott‐Schottky heterojunction improved the charge transfer kinetics of the material, while the built‐in electric fields formed at the heterointerfaces of CoS to CoS_2_ enhanced the Na^+^ diffusion kinetics. Thereby improving the sodium storage performance of the material.^[^ ^63^ ^]^ The inclusion of a graphene‐like carbon layer significantly reduces the diffusion resistance of Na^+^ and enhances the conductivity.^[^ ^64^ ^]^ Moreover, heterostructures exhibited superior diffusion capabilities compared to individual building blocks, suggesting the advantages of heterostructures and the presence of an effective electric field, leading to an improved rate of performance.^[^ ^65^ ^]^

In summary, the combined features of HC@CoS_2_/CoS/NC, such as improved electronic/ionic conductivity and reduced diffusion energy barrier, are theoretically supported by DFT calculations. These calculations additionally highlight that the formation of heterostructures, self‐induced electric fields, and electronic structure construction are crucial for improving charge/ion transport in SIBs, which ultimately results in improved Na^+^ storage.

### 2.5 Mechanistic Analysis of the HC@CoS_2_/CoS/NC Anode for Na+ Storage

**Figure 6:**
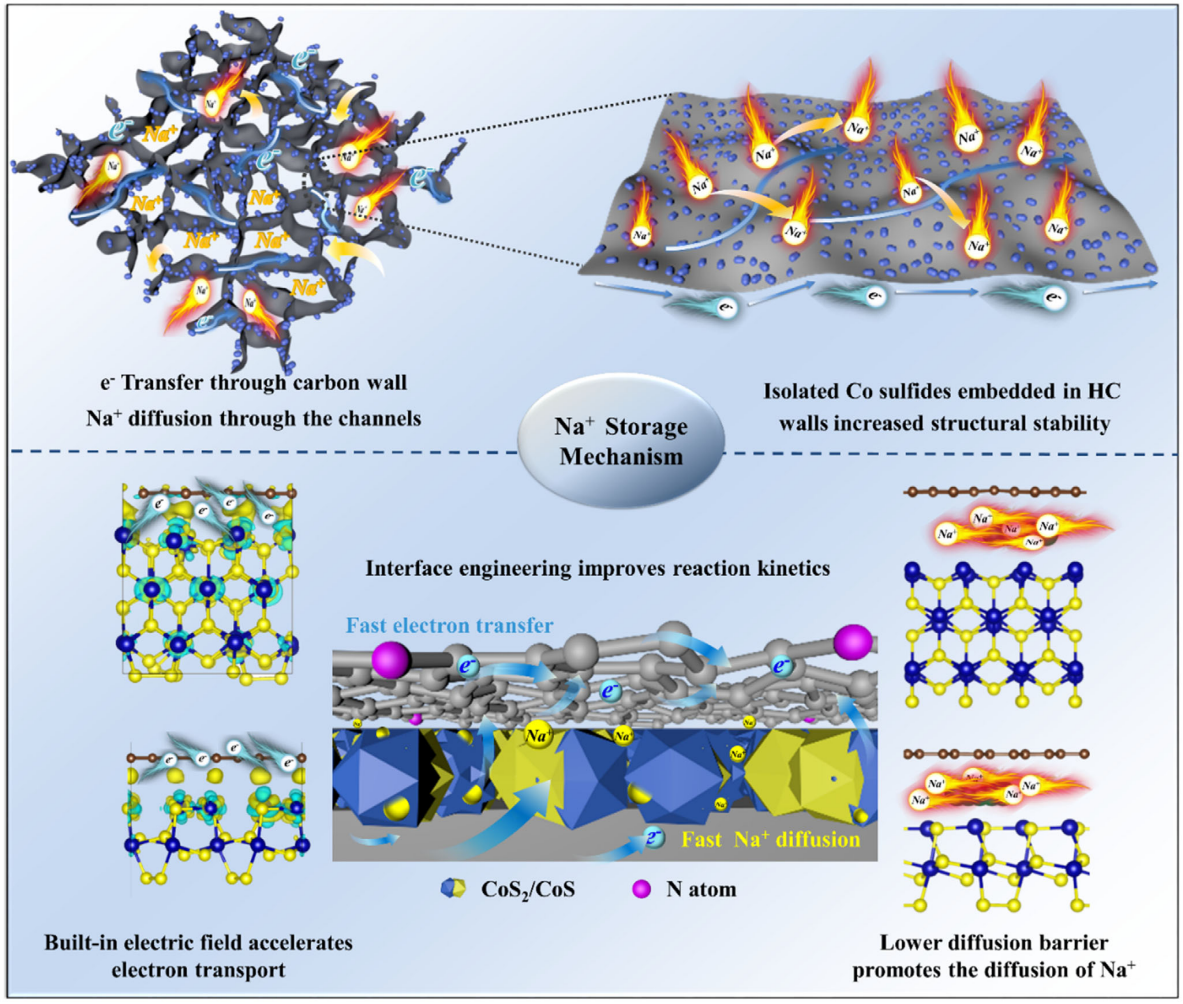
Schematic of HC@CoS_2_/CoS/NC for Na^+^ storage.

Based on the test results, a potential mechanism for the enhancement of the Na^+^ storage performance by HC@CoS_2_/CoS/NC was proposed in **Figure** **6**. In general, the unique structure and composition of HC@CoS_2_/CoS/NC provides several advantages for Na^+^ storage. The 3D HC offers extensive honeycomb walls for rapid electron transport and facilitates electrolyte diffusion into the interior electrodes, reducing the Na^+^ diffusion distance; secondly, the strong binding interaction between the cobalt sulfides and graphene‐like honeycomb walls significantly improves the structural stability by effectively preventing electrode pulverization and cobalt sulfide nanoparticle agglomeration while accommodating volume variation during charge/discharge cycles; Thirdly, heterojunction structure design improves the reaction kinetics and charge redistribution within the heterostructures formed between cobalt sulfides and carbon layers, leading to increased charge transfer, promoting the intrinsic electronic conductivity and accelerating electron transfer and the kinetic pathway of the conversion reaction through the built‐in electric field effect; additionally, the porous surface exposed oxygen‐containing functional groups from HC and N‐doping creates numerous additional reactive sites for Na^+^ storage, significantly enhancing the Na^+^ pseudocapacitive storage effect and increasing the reversible capacity and fast charge capability.

### 2.6 Full Battery Performance

**Figure 7:**
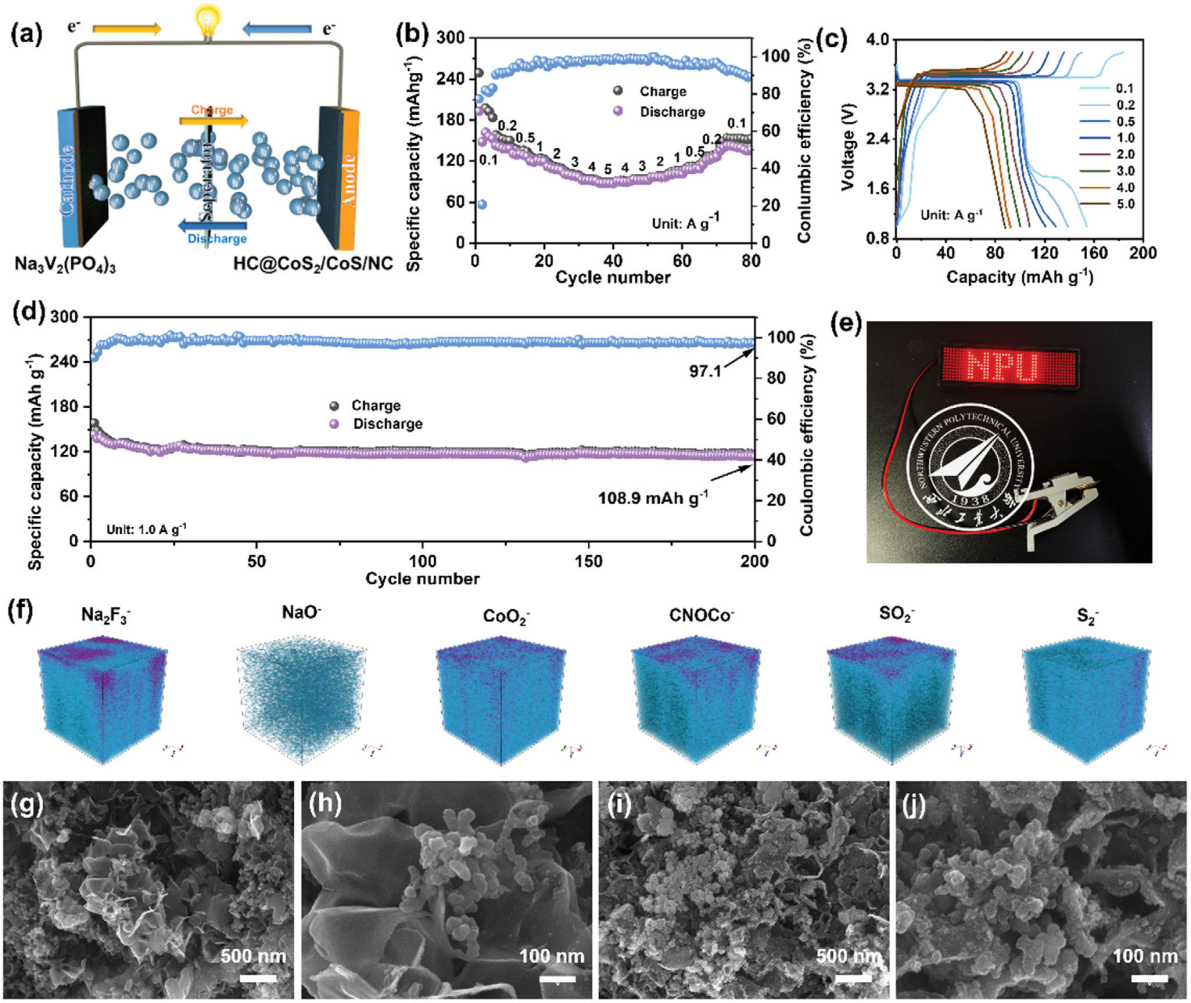
HC@CoS_2_/CoS/NC//NVP full battery: a) schematic illustration of the full battery, b) the rate capacities, c) the corresponding representative discharge‐charge curves, d) the cycle performance and e) illustration of the coin cell device powering the display board with the letter “NPU”; f) TOF‐SIMS 3D plots for Na_2_F_3_^−^, NaO^−^, CoO_2_^−^, CNOCo^−^, SO_2_^−^ and S_2_^−^. The postmortem SEM images of HC@CoS_2_/CoS/NC after g,h) 20 and i,j) 70 cycles at 1 A g^−1^.

Based on the outstanding Na^+^ storage properties observed in half‐cells, the practical application of HC@CoS_2_/CoS/NC in Na^+^ full‐cells devices with commercial Na_3_V_2_(PO_4_)_3_ (NVP) as the cathode was further assessed. The operational mechanism of the full battery is illustrated in **Figure** **7**a. The basic details regarding NVP were provided in Figure S14 (Supporting Information), displaying that NVP exhibited a well‐organized and well‐crystallized spherical morphology. The Na^+^ storage capabilities of the NVP cathode in the half‐cells are illustrated in Figure S15 (Supporting Information). The NVP cathode exhibited an excellent rate performance and cycling stability, achieving a high specific capacity of 98.6 mAh g^−1^ at 1.0 Ag^−1^ after 150 cycles.

Before assembly into the full cell, both the anode and cathode (Figure 7a) were subjected to an activation process for three cycles to mitigate the initial irreversible capacity loss and enhance the stability of the full cell during cycling. The full cell (Figure 7b) was tested within the voltage range of 1.0–3.8 V. The excellent rate performance and capability of the HC@CoS_2_/CoS/NC//NVP full cell to support rapid charging and discharging are illustrated in Figure 7c. As current densities increased to 0.1, 0.2, 0.5, 1.0, 2.0, 3.0, 4.0, and 5.0 A g^−1^, the average reversible capacities were measured at 154.14, 138.69, 128.73, 120.78, 107.76, 100.38, 92.26, and 87.93 mAh g^−1^, respectively (normalized by the active mass of the cathode and consistent with practices in other studies).^[^ ^66^ ^]^ The notable cycling performance is shown in Figure 7d. The full cell exhibited an initial reversible capacity of 108.90 mAh g^−1^ after 200 cycles at 1.0 A g^−1^ with a capacity retention of 80.81%. Additionally, the energy output of the coin‐type HC@CoS_2_/CoS/NC//NVP full cell was adequate to power a display board with the letter “NPU” made from light‐emitting diodes (LEDs) (Figure 7e), further confirming that HC@CoS_2_/CoS/NC holds significant promise as an anode material for practical sodium‐ion battery applications.

To gain more insights into the spatial arrangement of the SEI layer, time of‐flight secondary‐ion mass spectrometry (TOF‐SIMS) was employed to analyze the depth‐resolved compositional features on the HC@CoS_2_/CoS/NC anode. Figure 7f demonstrates that the primary components of the solid electrolyte interphase (SEI) layer formed on the anode surface are sodium fluoride (NaF) and sodium oxide (Na₂O). CoO_2_^−^ and CNOCo^−^ reveal that cobalt elements in the hierarchical carbon‐encapsulated CoS₂/CoS embedded in nitrogen‐doped carbon matrix (HC@CoS_2_/CoS/NC) chemically interact with organic fragments generated from electrolyte decomposition during charge/discharge cycles, resulting in uniform SEI deposition. The detected sulfur signal may originate from either electrolyte decomposition or the intrinsic CoS_2_/CoS components. SO_2_^−^ and S_2_^−^ further confirm the homogeneous distribution of sulfur species across the electrode interface, collectively indicating the formation of a robust and stable SEI architecture. This structural homogeneity significantly contributes to enhanced electrochemical stability by suppressing localized ion flux fluctuations and mechanical stress accumulation.

## 3 Conclusion

In summary, a novel HC@CoS_2_/CoS/NC composite with optimized heterointerfaces was successfully synthesized as an ultrastable and fast‐charging anode material for SIBs. This composite features a 3D architecture, characterized by highly dispersed CoS_2_/CoS/NC nanoparticles embedded within honeycomb carbon nanosheets. Honeycomb carbon materials with extensive surfaces and oxygen‐containing functional groups have been utilized as both active substrates and conductive platforms, significantly enhancing the overall conductivity. Furthermore, HC@CoS_2_/CoS/NC offers numerous heterogeneous interfaces for Na^+^ migration and enhances electrochemical kinetics. The carefully designed hierarchical nanostructures provided stress buffering and facilitated ion transport, effectively extending the cycling lifespan. The HC@CoS_2_/CoS/NC composites demonstrated remarkable Na^+^ storage capability, high reversible capacity, and superior prolonged cycling ability (465.2 mAh g^−1^ for 700 cycles at 15 A g^−1^), making them highly competitive in fast‐charging applications. In addition, when coupled with an NVP cathode, the full battery maintained a specific capacity of 108.9 mAh g^−1^ after 200 cycles at 1.0 A g^−1^.

## 4 Experimental Section

All reagents were of analytical grade and were used without further purification.

### Synthesis of Honeycomb‐Like Carbon (HC)

Honeycomb‐like carbon was prepared according to a previous study.^[^ ^24^ ^]^ Typically, trisodium citrate dihydrate (Na_3_C_6_H_5_O_7_·2H_2_O) was carbonized at 600 °C for 2 h under argon atmosphere at a heating rate of 5 °C min^−1^. After cooling to ambient temperature, a black powder was obtained. The black powder was then submerged in dilute hydrochloric acid (4 mol L^−1^) for etching and removal of residual sodium carbonate, followed by thorough washing with deionized water to remove impurities. Subsequently, the samples were freeze‐dried for 12 h to obtain HC.

### Synthesis of HC@ZIF‐67 Composites

In a typical synthetic process, 0.02 g of HC and 1 mmol (0.291 g) of Co(NO_3_)_2_·6H_2_O were uniformly dispersed in ethanol by ultrasound. Subsequently, 8 mmol (0.657 g) of 2‐methylimidazole (2‐MIM) was added, and the mixture was maintained at 70 °C for 2 h. After cooling, the resulting purple‐black mixture was subjected to repeated centrifugation and washed with ethanol to remove any uncreated 2‐MIM. The purple‐black sediments were dried in an oven at 60 °C to obtain the HC@ZIF‐67 composites.

### Synthesis of HC@CoS_2_/CoS/NC Composites

0.10 g of the obtained HC@ZIF‐67 composites and 0.30 g of sulfur powder were placed at the upstream and downstream ends of the tubular furnace, respectively. The furnace was heated to 550 °C for 2 h in an Ar atmosphere at a heating rate of 2 ^°^C ·min^−1^. The remaining black powder was designated as the HC@CoS_2_/CoS/NC composite.

For comparison, the CoS_2_/CoS/NC samples were obtained using the same procedure by replacing HC@ZIF‐67 with ZIF‐67.

## Conflict of Interest

The authors declare no conflict of interest.

## Supporting information



Supporting Information

## Data Availability

The data that support the findings of this study are available from the corresponding author upon reasonable request.

## References

[1] H. Wu , K. Wang , M. Li , Y. Wang , Z. Zhu , JialeLiang, Z. D.u , W. Ai , S. He , R. Yuan , B. Wang , P. He , J. Wu , Small 2023, 19, 2300162. 10.1002/smll.202300162 36866502

[2] J. A. S. Oh , G. Deysher , P. Ridley , Y.‐T. Chen , D. Cheng , A. Cronk , S.‐Y. Ham , D. H. S. Tan , J. Jang , L. H. B. Nguyen , Y. S. Meng , Adv. Energy Mater. 2023, 13, 2300776.

[3] S. You , Q. Zhang , J. Liu , Q. Deng , Z. Sun , D. Cao , T. Liu , K. Amine , C. Yang , Energy Environ. Sci. 2024, 17, 8189.

[4] C. Yang , W. Zhong , Y. Liu , Q. Deng , Q. Cheng , X. Liu , C. Yang , Carbon Energy 2024, 6, 503.

[5] Y. V. Lim , X. L. Li , H. Y. Yang , Adv. Funct. Mater. 2021, 31, 2006761.

[6] C.‐Y. Wang , T. Liu , X.‐G. Yang , S. Ge , N. V. Stanley , E. S. Rountree , Y. Leng , B. D. McCarthy , Nature 2022, 611, 485. 36224388 10.1038/s41586-022-05281-0

[7] L. Cao , X. Liang , X. Ou , X. Yang , Y. Li , C. Yang , Z. Lin , M. Liu , Adv. Funct. Mater. 2020, 30, 1910732.

[8] G.‐L. Zhu , C.‐Z. Zhao , J.‐Q. Huang , C. He , J. Zhang , S. Chen , L. Xu , H. Yuan , Q. Zhang , Small 2019, 15, 1805389. 10.1002/smll.201805389 30869836

[9] D. Wu , Y. Kang , F. Wang , J. Yang , Y. Xu , Y. Zhuang , J. Wu , J. Zeng , Y. Yang , J. Zhao , Adv. Energy Mater. 2023, 13, 2301145.

[10] Y. Liu , X. Hu , J. Li , G. Zhong , J. Yuan , H. Zhan , Y. Tang , Z. Wen , Nat. Commun. 2022, 13, 663. 35115491 10.1038/s41467-022-28176-0 PMC8814252

[11] Y. Gan , L. Liu , Q. Zhang , J. Huang , S. Han , B. Chen , Y. Liu , Q. Yu , L. Guan , T. Zhou , M. Han , Y. Zhao , W. Huang , Energy Stor. Mater. 2023, 59, 102794.

[12] X. Ou , L. Cao , X. Liang , F. Zheng , H. S. Zheng , X. Yang , J. H. Wang , C. Yang , M. Liu , ACS Nano 2019, 13, 3666. 30785716 10.1021/acsnano.9b00375

[13] X. Li , H. Liang , B. Qin , M. Wang , Y. Zhang , H. Fan , J. Colloid Interface Sci. 2022, 625, 41. 35714407 10.1016/j.jcis.2022.05.155

[14] Q. Li , Q. Jiao , Y. Yan , H. Li , W. Zhou , T. Gu , X. Shen , C. Lu , Y. Zhao , Y. Zhang , H. Li , C. Feng , Chem. Eng. J. 2022, 450, 137922.

[15] C. Yang , X. Liang , X. Ou , Q. Zhang , H. S. Zheng , F. Zheng , J. H. Wang , K. Huang , M. Liu , Adv. Funct. Mater. 2019, 29, 1807971.

[16] Y. Yuan , Z. Chen , H. Yu , X. Zhang , T. Liu , M. Xia , R. Zheng , M. Shui , J. Shu , Energy Stor. Mater. 2020, 32, 65.

[17] G. Wan , W. Dou , H. Zhu , W. Zhang , T. Liu , L. Wang , J. Lu , Interd. Mater 2023, 2, 416.

[18] Q. Wei , T. Huang , X. Huang , B. Wang , Y. Jiang , D. Tang , D. L. Peng , B. Dunn , L. Mai , Interd. Mater 2023, 2, 434.

[19] H. Li , Y. Y. He , Q. Wang , S. A. Gu , L. Wang , J. X. Yu , G. W. Zhou , L. Q. Xu , Adv. Energy Mater. 2023,13, 2302901.

[20] M. Yang , X. Chang , L. Wang , X. Wang , M. Gu , H. Huang , L. Tang , Y. Zhong , H. Xia , Adv. Mater. 2023, 35, 2208705. 10.1002/adma.202208705 36661129

[21] S. Y. Liao , J. Chen , S. F. Cui , J. Q. Shang , Y. Z. Li , W. X. Cheng , Y. D. Liu , T. T. Cui , X. G. Shu , Y. G. Min , J. Power Sources 2023, 553, 232265.

[22] Z. Kong , L. Wang , S. Iqbal , B. Zhang , B. Wang , J. M. Dou , F. B. Wang , Y. T. Qian , M. Zhang , L. Q. Xu , Small 2022, 18, 2107252. 10.1002/smll.202107252 35224841

[23] C. Shen , G. Song , X. Zhu , D. Wang , L. Huang , Z. Sun , Y. Wu , Nano Energy 2020, 78, 105294.

[24] N. Cheng , W. Zhou , J. Liu , Z. Liu , B. Lu , Nano‐Micro Lett. 2022, 14, 146. 10.1007/s40820-022-00892-8 PMC9304482 35861905

[25] B. Yang , J. Chen , S. Lei , R. Guo , H. Li , S. Shi , X. Yan , Adv. Energy Mater. 2018, 8, 1702409.

[26] H. Cui , J. Zheng , P. Yang , Y. Zhu , Z. Wang , Z. Zhu , ACS Appl. Mater. Interfaces 2015, 7, 11230. 25961810 10.1021/acsami.5b01201

[27] L.‐B. Ren , W. Hua , Z.‐D. Hou , J.‐G. Wang , Rare Met. 2022, 41, 1859.

[28] T. Zhang , F. Ran , Adv. Funct. Mater. 2021, 31, 2010041.

[29] F. Sun , H. Wang , Z. Qu , K. Wang , L. Wang , J. Gao , J. Gao , S. Liu , Y. Lu , Adv. Energy Mater. 2021, 11, 2002981.

[30] J.‐L. Xia , D. Yan , L.‐P. Guo , X.‐L. Dong , W.‐C. Li , A.‐H. Lu , Adv. Mater. 2020, 32, 2000447. 10.1002/adma.202000447 32253798

[31] H. Pan , Z. Cheng , J. Fransaer , J. Luo , M. Wübbenhorst , Nano Res. 2022, 15, 8091.

[32] F. Wu , V. Srot , S. Chen , S. Lorger , P. A. van Aken , J. Maier , Y. Yu , Adv. Mater. 2019, 31, 1905146. 10.1002/adma.201905146 31513323

[33] D. Ma , B. Hu , W. Wu , X. Liu , J. Zai , C. Shu , T. Tadesse Tsega , L. Chen , X. Qian , T. L. Liu , Nat. Commun. 2019, 10, 3367. 31358738 10.1038/s41467-019-11176-y PMC6662769

[34] X. Hu , P. Tan , R. Dong , M. Jiang , L. Lu , Y. Wang , H. Liu , Y. Liu , J. Xie , J. Pan , Energy Technol. 2021, 9, 2000961.

[35] W. Tan , L. Wang , K. Liu , Z. Lu , F. Yang , G. Luo , Z. Xu , Small 2022, 18, 2203494. 10.1002/smll.202203494 36029270

[36] Q. Huang , C. Wang , Q. Shan , Nanomaterials 2022, 12, 2320.35889545

[37] Z. Zhao , X. Pei , J. Li , Y. Qin , C. Li , J. Cheng , Y. Fu , X. Du , D. Li , Energy Environ. Mater. 2022, 6, e12467.

[38] Y. Wu , J. Cheng , Z. Liang , T. Qiu , Y. Tang , J. Shi , S. Gao , R. Zhong , R. Zou , Carbon 2022, 198, 353.

[39] J. Tang , J. Shen , N. Li , M. Ye , Ceram. Int. 2014, 40, 15411.

[40] L. Vála , R. Medlín , M. Koštejn , S. Karatodorov , V. Jandová , V. Vavruňková , T. Křenek , Eur. J. Inorg. Chem. 2019, 2019, 1220.

[41] Y. Zhan , S.‐z. Yu , S.‐h. Luo , J. Feng , Q. Wang , ACS Appl. Mater. Interfaces 2021, 13, 17658. 33826308 10.1021/acsami.1c02564

[42] S. Jing , C. Y. Zhao , X. J. Zhang , S. Kong , X. Lan , Z. P. Ma , H. Feng , W. B. Gong , K. H. Tian , Q. L. Li , Y. B. Feng , Adv. Mater. Interfaces 2022, 9, 2201230.

[43] S. Li , J. Qin , T. Gao , J. Du , K. Yuan , N. Li , L. Xu , J. Xu , J. Alloys Compd. 2022, 912, 165130.

[44] J. Xu , P. Ye , Y. Cheng , L. Ji , Y. Wei , Y. Chen , Energy Technol. 2023, 11, 2201452.

[45] L. Kong , Y. Liu , H. Huang , M. Liu , W. Xu , B. Li , X.‐H. Bu , Sci. China Mater. 2021, 64, 820.

[46] S. H. Yang , S.‐K. Park , Y. C. Kang , Nano‐Micro Lett. 2020, 13, 9. 10.1007/s40820-020-00539-6 PMC8187686 34138196

[47] J. Chen , Z. Zhang , H. Li , J. Electroanal. Chem. 2022, 911, 116203.

[48] Y. Wang , Y. Zhang , Y. Peng , H. Li , J. Li , B.‐J. Hwang , J. Zhao , Electrochim. Acta 2019, 299, 489.

[49] Q. Shi , K. Chen , Z. Yu , M. Fang , Z. Dai , J. Wang , K. Cao , F. Tian , Y. Zhang , S. Yang , X. Zhou , J. Alloys Compd. 2022, 902, 163812.

[50] X. Zhang , F. Wu , X. Lv , L. Xu , R. Huang , R. Chen , L. Li , Adv. Mater. 2022, 34, 2204370. 10.1002/adma.202204370 35973233

[51] Z. Zhang , Y. Huang , X. Gao , Z. Xu , X. Wang , ACS Appl. Energy Mater. 2020, 3, 6205.

[52] B. Yin , X. Cao , A. Pan , Z. Luo , S. Dinesh , J. Lin , Y. Tang , S. Liang , G. Cao , Adv. Sci. 2018, 5, 1800829. 10.1002/advs.201800829 PMC6145217 30250811

[53] H. Liu , P. He , L. Jia , M. He , X. Zhang , S. Wang , X. Zhang , C. Li , Y. Zhang , F. Dong , Ionics 2018, 24, 3591.

[54] C. Choi , D. S. Ashby , D. M. Butts , R. H. DeBlock , Q. Wei , J. Lau , B. Dunn , Nat. Rev. Mater. 2020, 5, 5.

[55] S. H. Yang , S.‐K. Park , Y. C. Kang , Chem. Eng. J. 2019, 370, 1008.

[56] M. Xie , C. Li , S. Zhang , Z. Zhang , Y. Li , X.‐B. Chen , Z. Shi , S. Feng , Small 2023, 19, 2301436. 10.1002/smll.202301436 37078904

[57] Q. Li , Q. Jiao , W. Zhou , X. Feng , Q. Shi , Z. Dai , T. Gu , Y. Zhao , H. Li , C. Feng , Mater. Chem. Front. 2021, 5, 293.

[58] T. Zhang , Y. Feng , J. Zhang , C. He , D. M. Itkis , J. Song , Mater. Today Nano. 2020, 12, 100089.

[59] X. Li , H. Liang , B. Qin , M. Wang , Y. Zhang , H. Fan , J. Colloid Interf. Sci. 2022, 625, 41. 10.1016/j.jcis.2022.05.155 35714407

[60] X. Zhang , W. Weng , H. Gu , Z. Hong , W. Xiao , F. Wang , W. Li , D. Gu , Adv. Mater. 2022, 34, 2104427. 10.1002/adma.202104427 34676913

[61] W. Li , C. Liu , C. Gu , J.‐H. Choi , S. Wang , J. Jiang , J. Am. Chem. Soc. 2023, 145, 4774. 36802572 10.1021/jacs.2c13596

[62] J. Wang , X. Yue , X. Cao , Z. Liu , A. M. Patil , J. Wang , X. Hao , A. Abudula , G. Guan , Chem. Eng. J. 2022, 431, 134091.

[63] S. Dong , C. Li , Z. Li , L. Zhang , L. Yin , Small 2018, 14, 1704517. 10.1002/smll.201704517 29575525

[64] X. Hu , Y. Liu , J. Li , G. Wang , J. Chen , G. Zhong , H. Zhan , Z. Wen , Adv. Funct. Mater. 2020, 30, 1907677.

[65] Y. Li , M. Han , Z. Zhou , X. Xia , Q. Chen , M. Chen , ChemElectroChem 2021, 8, 697.

[66] J. Zhao , Y. Zhang , X. Chen , G. Sun , X. Yang , Y. Zeng , R. Tian , F. Du , Adv. Funct. Mater. 2022, 32, 2206531.

