# Supplementary material for: Enhanced Reaction Kinetics in Sodium‐Ion Batteries Achieved by 3D Heterostructure CoS2/CoS with Self‐Induced Internal Electric Field
Source: Adv Sci (Weinh). 2025 Apr 25;12(26):2502241. doi: 10.1002/advs.202502241 (PMC12244505; doi:10.1002/advs.202502241)
Supplement: Supplementary file 1 — Supporting Information [file ADVS-12-2502241-s001.docx]

**Supporting Information**

**Enhanced Reaction Kinetics in Sodium-ion Batteries Achieved by 3D Heterostructure CoS_2_/CoS with Self-induced Internal Electric Field**

Jin Liang, ^1, 2, 3^* Jiawen Sun, ^1^ Xin Cao,^1^ Xiaoshan Li^1^, Xiaoyi Chen^1^, Ruizhe Xing^1^ and Jie Kong ^1,^*

*^1^MOE Key Lab of Materials Physics and Chemistry in Extraordinary Conditions, Shaanxi Key Lab of Macromolecular Science and Technology, School of Chemistry and Chemical Engineering, Northwestern Polytechnical University, Xi’an, 710072, P.R. China*

*^2^Key laboratory of Flexible Electronics of Zhejiang Province, Ningbo Institute of Northwestern Polytechnical University, 218 Qingyi Road, Ningbo, 315103, P.R. China*

^3^*Research & Development Institute of Northwestern Polytechnical University in Shenzhen; Sanhang Science &Technology Building, No.45th, Gaoxin South 9th Road, Nanshan District, Shenzhen City, 518063, P.R. China*

*Corresponding Authors, E-mail: [jin.liang@nwpu.edu.cn](mailto:jin.liang@nwpu.edu.cn) (J.L.), [kongjie@nwpu.edu.cn](mailto:kongjie@nwpu.edu.cn) (J.K.)

**1.1. Materials characterization**

The crystallographic phases of the materials were determined by X-ray diffraction (XRD, Bruker D8 Advance). The morphologies and microstructures were observed using transmission electron microscopy (TEM, Talos F200X) and field-emission scanning electron microscopy (FE-SEM, Verios G4) with energy dispersive spectroscopy (EDS). Raman spectra were acquired using Raman spectrometer (Alpha300R, WITec) with an argon laser at excitation wavelength of 532 nm. Besides, surface elements were investigated through X-ray photoelectron spectroscopy (XPS, Kratos Axis Ultra DLD). The binding energies of the samples were calibrated using the C 1s peak as a reference (284.8 eV). Fourier transform infrared (FT-IR) spectra were collected using an FT-IR spectrometer (IRTracer-100, Shimadu, Japan). The specific surface area and pore size distribution were tested using the Brunauer–Emmett–Teller method (BET, BeiShiDe3H-2000PS2, China) in N_2_ at 77 K.

**1.2. Electrochemical measurements**

The electrochemical behavior of all the materials was investigated using CR2016 coin-type cells assembled in an Ar-filled glove box (Mikrouna, Germany, [O_2_]<0.1 ppm, [H_2_O]<0.1 ppm). The working electrode was prepared by compressing a mixture of active materials, conductive material (Super P), and binder (polyvinylidene fluoride, PVDF) in a weight ratio of 70:20:10 and pasted on a Cu foil current collector. The electrode was pre-dried in an oven at 80 °C for 4 h and then thoroughly dried overnight at 120 °C under vacuum. Sodium slices were applied as the counter and reference electrodes, and the electrolyte used was 1.0 M NaPF_6_ in DME was used as the electrolyte. Glass fiber from Whatman (GF/D) was adopted as the separator. Galvanostatic charge-discharge behavior tests were carried out using a NEWARE multichannel battery system with a voltage range of 0.01-3.0 V at ambient temperature. Cyclic voltammetry measurements were performed on an electrochemical workstation (CHI 760E, CH Instruments, Shanghai) over a potential range of 0.01-3.0 V versus. Na/Na^+^ at a scan rate of 0.1 mV s^−1^. Electrochemical impedance spectroscopy was performed on the same workstation over the frequency range of 0.01 Hz-100 KHz. For the active nanocomposite electrodes, all the reported specific capacities and current densities were based on the total weight of the nanocomposite. The full battery was assembled using Na_3_V_2_(PO_4_)_3_ as the cathode with an N/P ratio of approximately 1.1.

**1.3. Density functional theory simulation**

Spin-polarized density functional theory computations were performed using the Vienna Ab initio simulation package code. The interaction between ions and electrons was described by the projector-augmented wave mode with the frozen-core approximation. The Kohn-Sham valence states were stretched in a plane-wave basis set with a kinetic cutoff energy of 400 eV. The exchange-correlation interaction was investigated using the spin-polarized Perdew–Burke–Ernzerhof exchange-correlation functional within generalized gradient approximation calculations. For the Brillouin zone integration, structure optimization was performed through a Monkhorst-Pack 3×3×1 mesh, whereas a 5×5×1 k-point was employed for electronic structure calculations. The van der Waals interaction was studied using the dispersion correction approach DFT-D3 with Becke–Johnson damping. All the configurations were completely relaxed until the forces on every atom were smaller than 0.05 eV Å^-1^. The convergence criterion of the self-consistent field was set at 1×10^-4^ eV. The DFT + U method was employed for Co with a value of U= 3.0 eV. The climbing image nudged-elastic band method was used to determine the diffusion energy barriers of Na^+^ ions. Charge density difference (CDD, Δ*ρ*) plots were achieved by the following equation:

$$\Delta\rho=\rho_{\mathrm{AB}}-\rho_{A}-\rho_{B}$$

where$\rho_{\mathrm{AB}}$representss the charge densities of the entire system;$\rho_{A}$and$\rho_{B}$ were the charge density of the two subsystems.

**
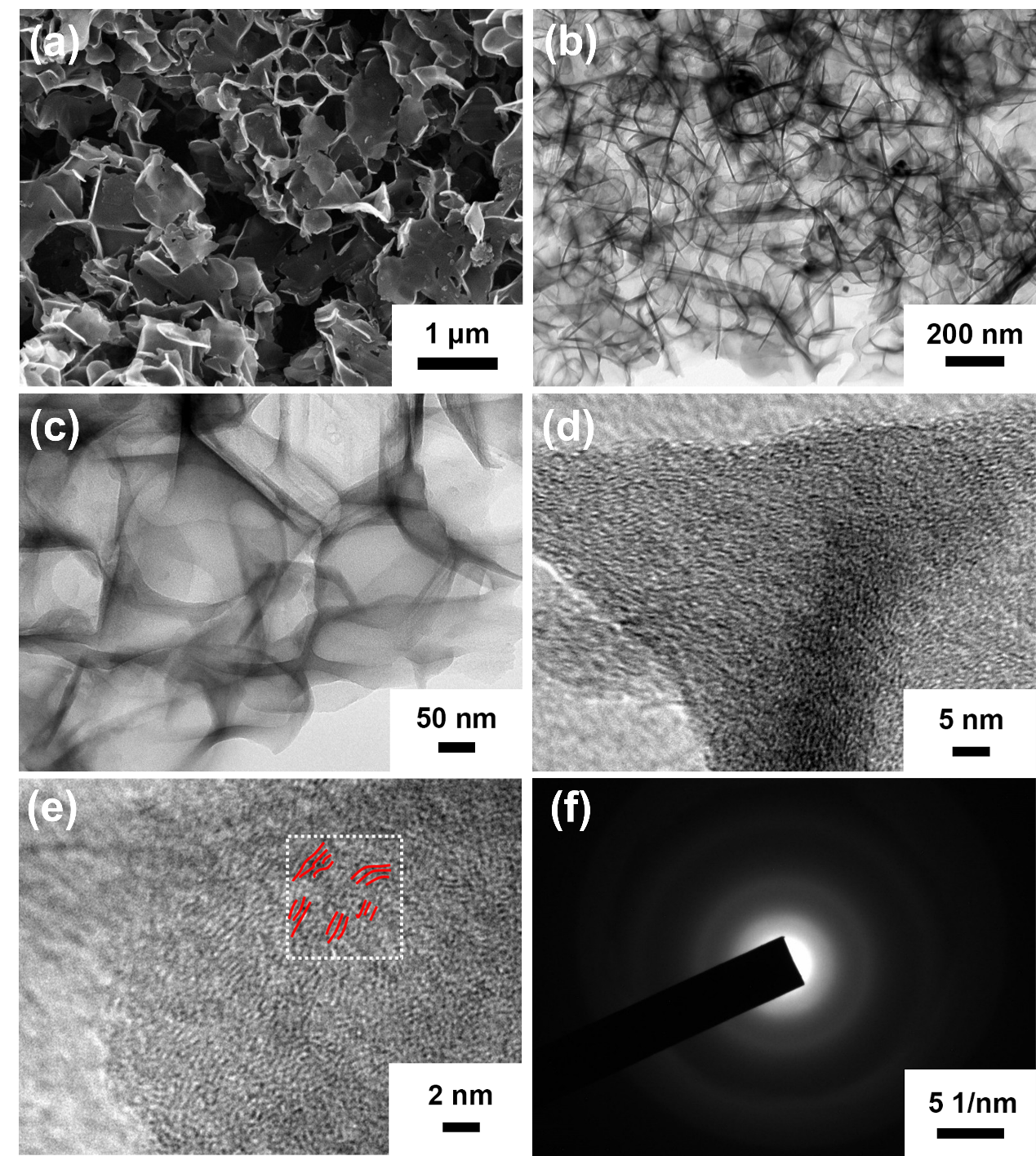
**

**Figure S1** (a) SEM, (b-e) TEM and (f) SAED patterns of HC.


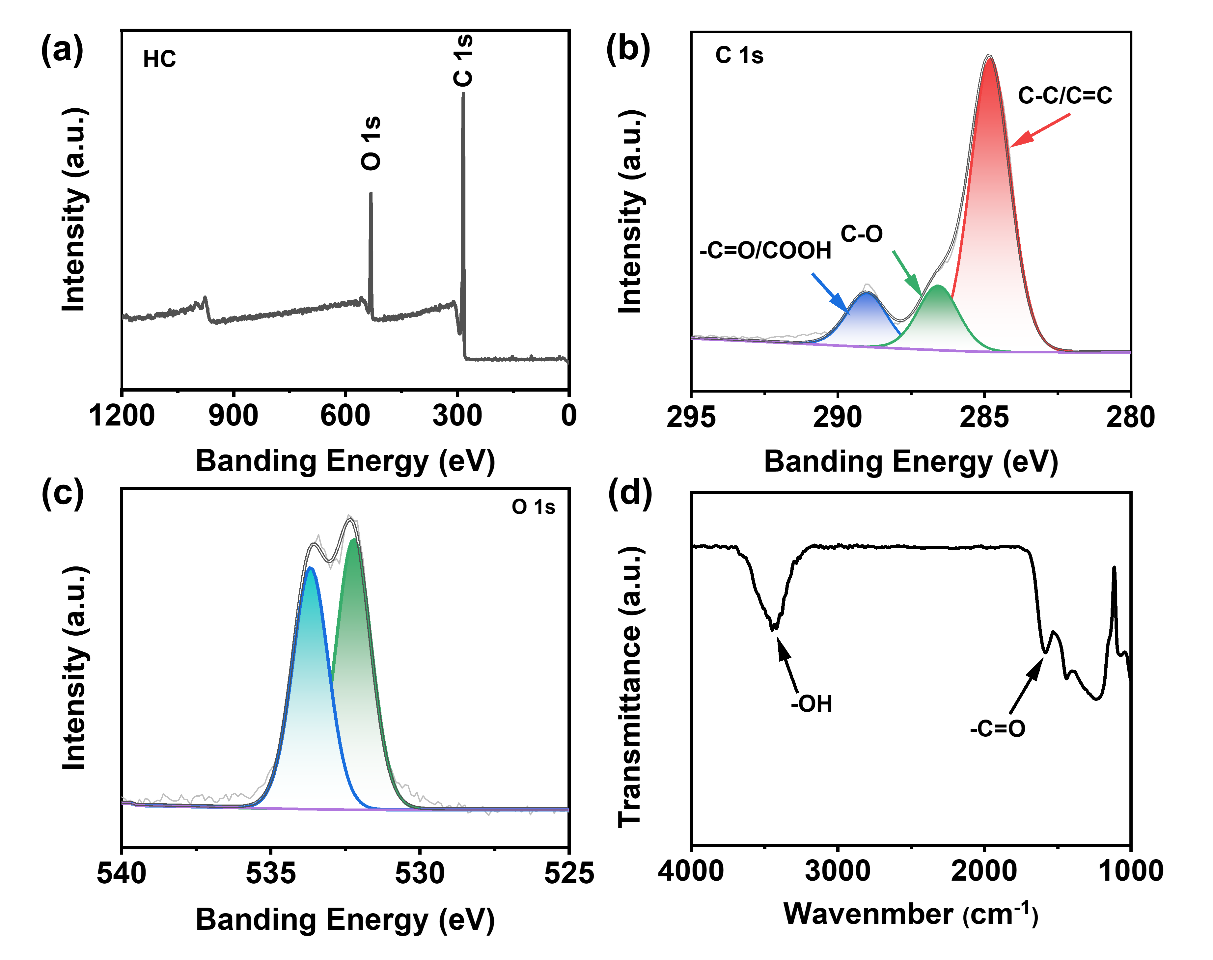


**Figure S2** (a) XPS scan spectrum, high-resolution XPS spectra of (B) C 1s and (c) O 1s, and (d) FT-IR spectrum of HC.

**
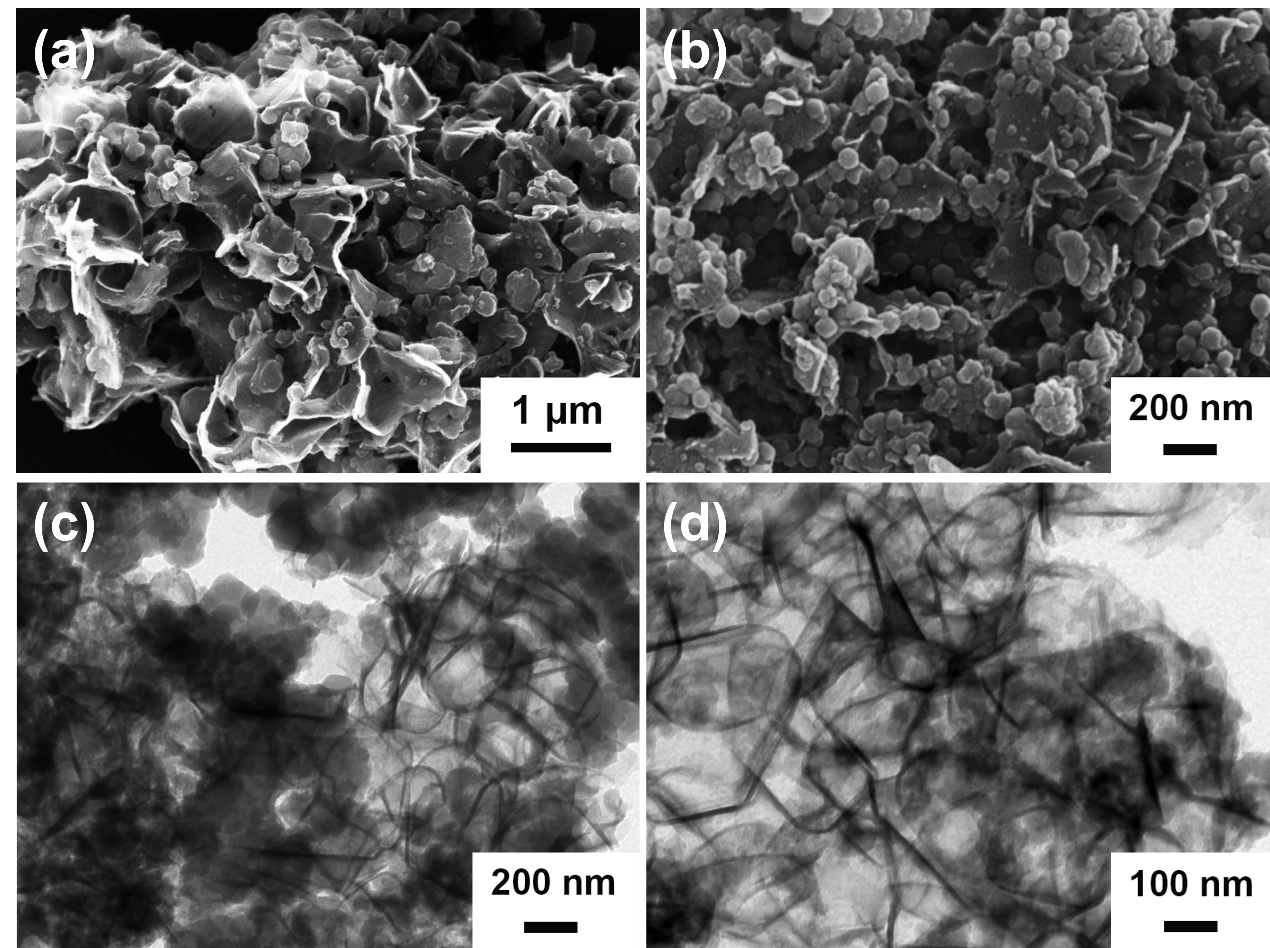
**

**Figure S3** (a-b) SEM and (c-d) TEM images of HC@ZIF-67.


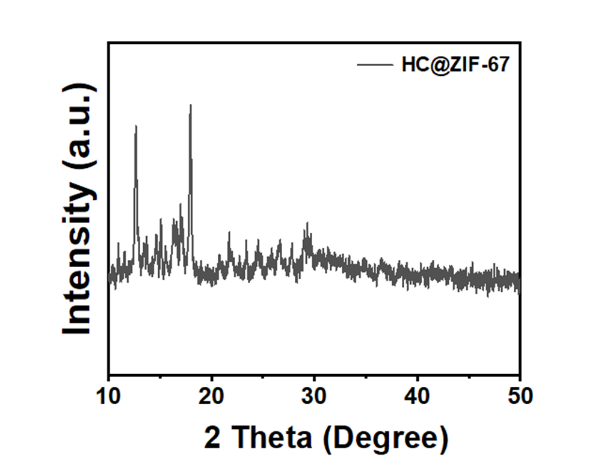


**Figure S4** XRD pattern of the HC@ZIF-67.


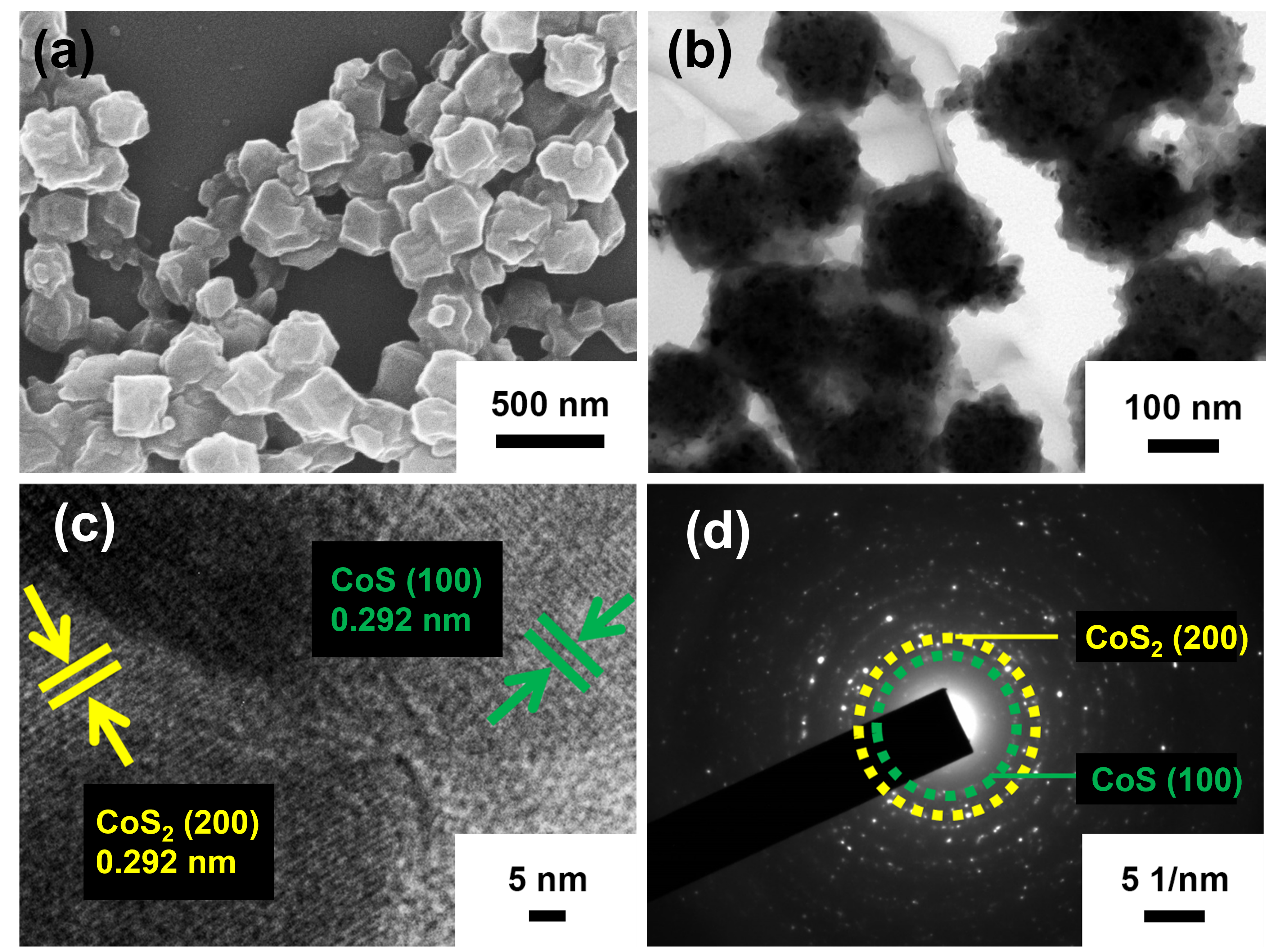


**Figure S5** CoS_2_/CoS/NC: (a) SEM, (b) TEM, (c) HRTEM, and (d) SAED images.


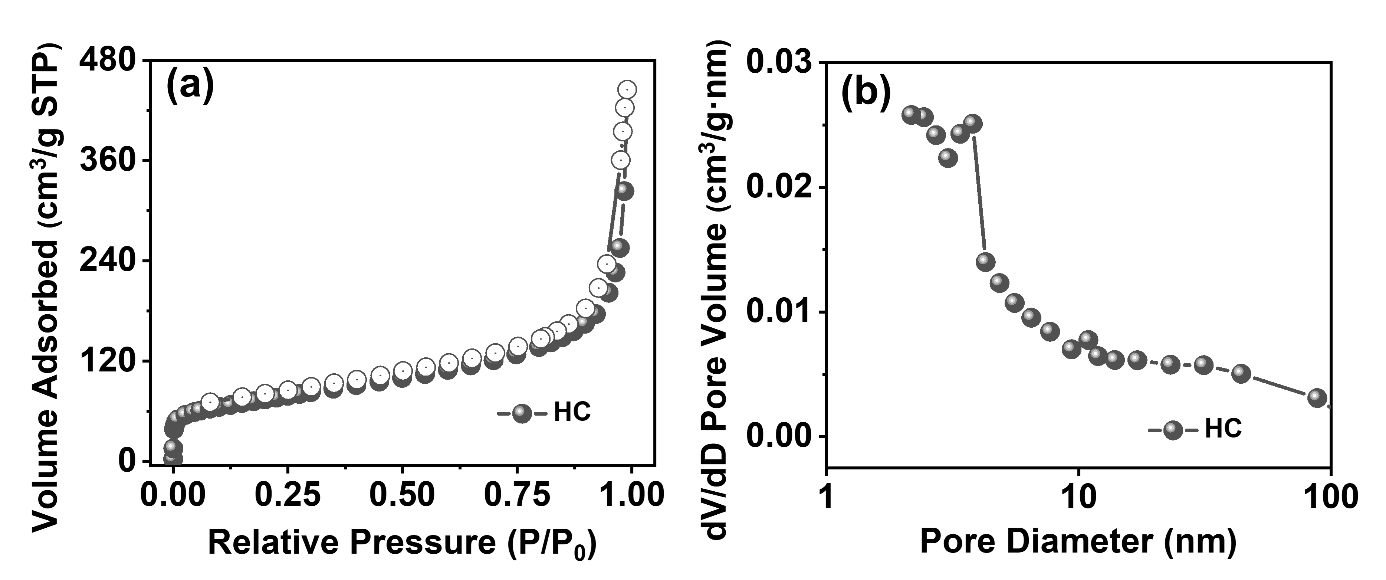


**Figure S6** (a) N_2_ adsorption/desorption curve and (b) pore diameter distribution of HC.

**Table S1** Specific surface areas, total pore volumes, and BHJ pore diameters of three samples.

| **Sample** | HC | HC@CoS_2_/CoS/NC | CoS_2_/CoS/NC |
| --- | --- | --- | --- |
| **BET Surface Area (m^2^ g^-1^)** | 262.80 | 39.93 | 21.86 |
| **Total pore volume (cm^3^ g^-1^)** | 0.688 | 0.221 | 0.110 |
| **BJH pore diameter (nm)** | 3.802 | 3.793 | 3.796 |

**
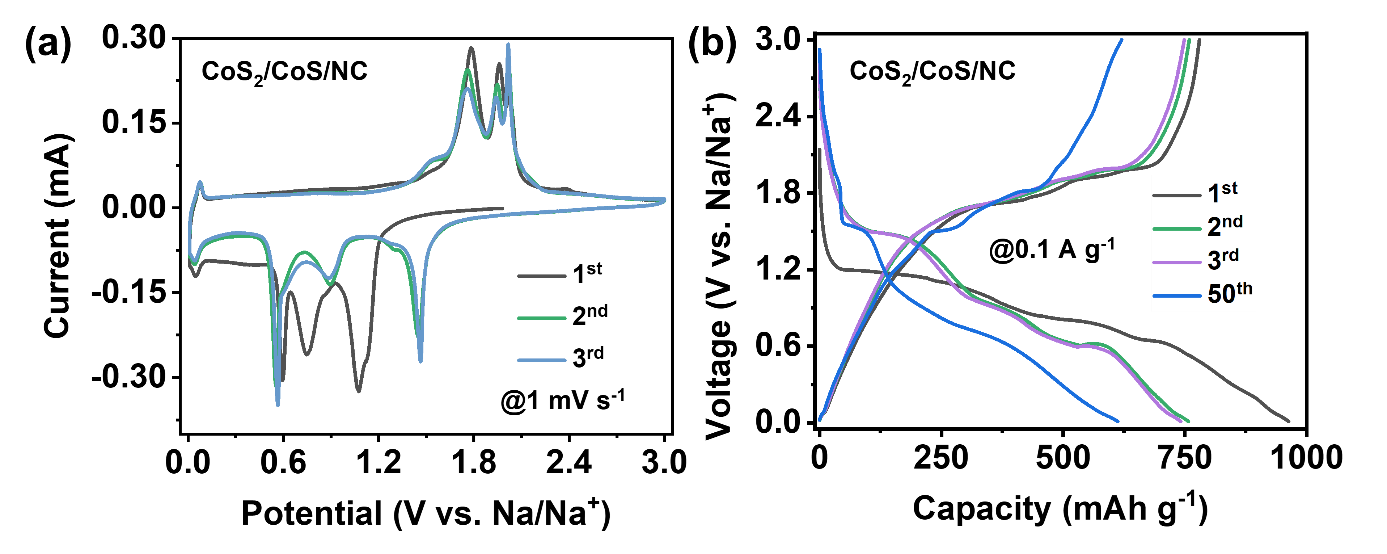
**

**Figure S7** (a) CV curves at the scan rate of 0.1 mV s^−1^ and (b) charge-discharge curves at 0.1 A g^−1^ of CoS_2_/CoS/NC.

**
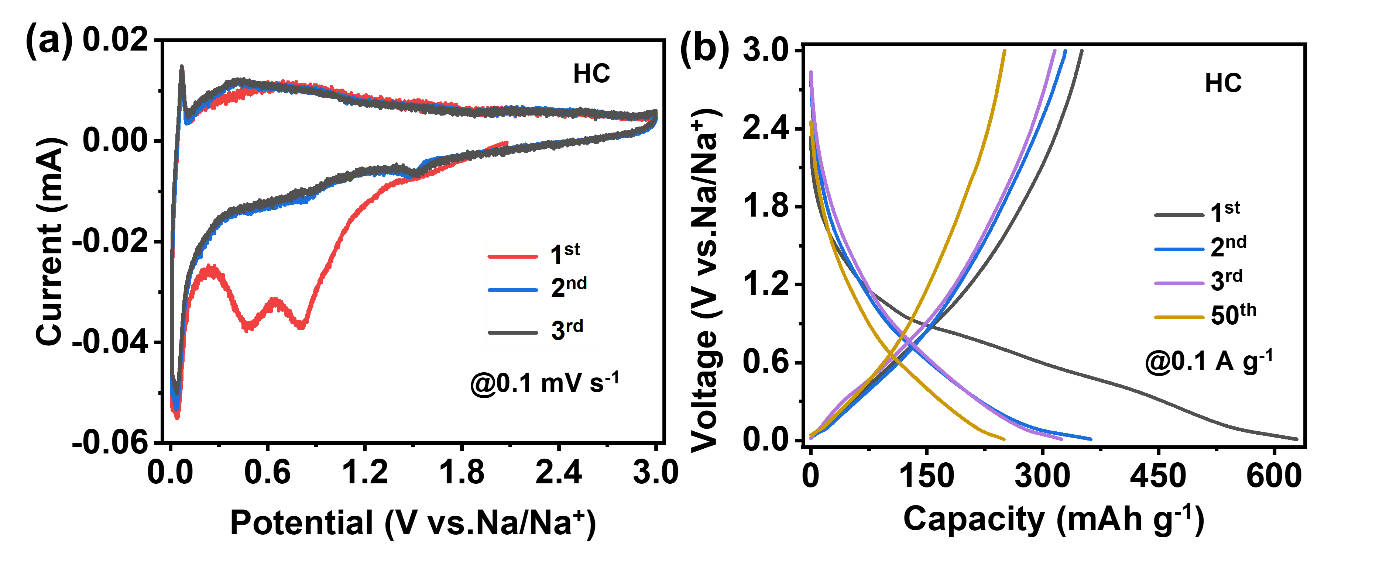
**

**Figure S8 (**a) CV curves at the scan rate of 0.1 mV s^−1^ and (b) charge-discharge at 0.1 A g^−1^ curves of HC.

**
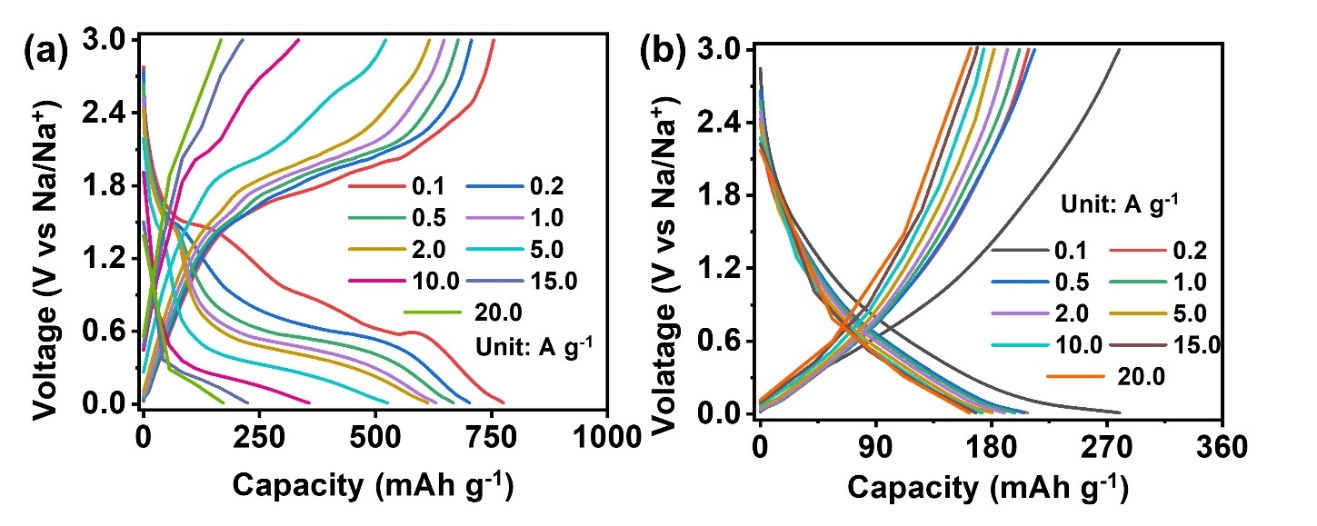
**

**Figure S9** Charge and discharge profiles at different current densities of (a) CoS_2_/CoS/NC and (b) HC electrode.


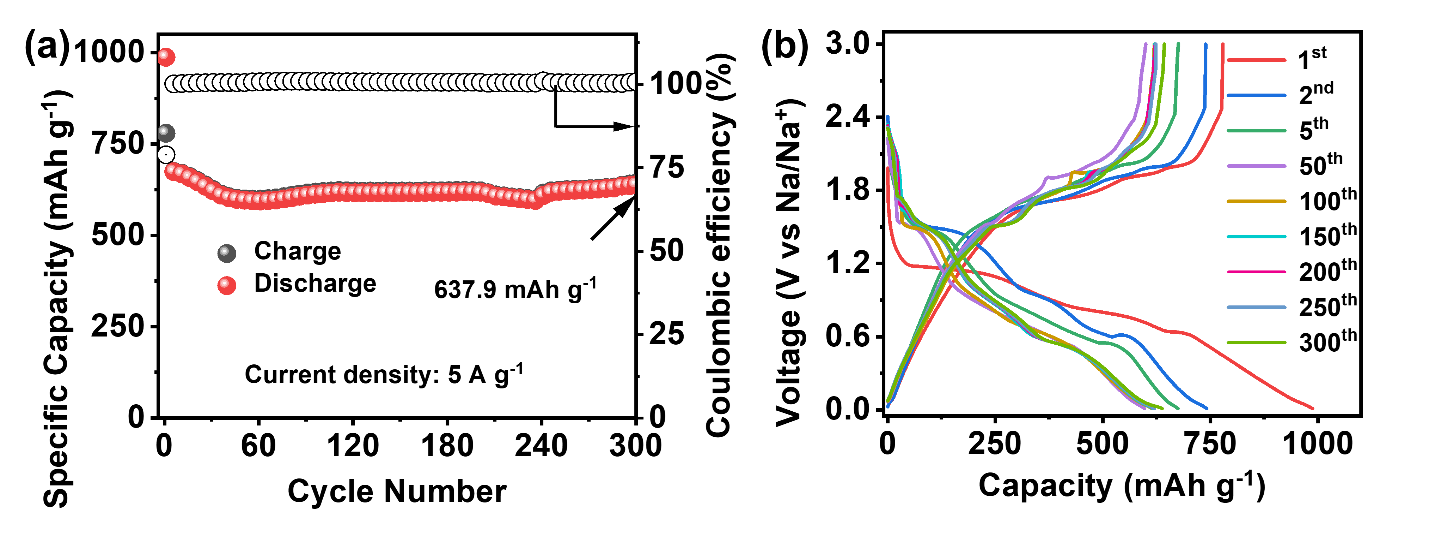


**Figure S10** (a) Cycle performance, and (b) charge and discharge curves of HC@CoS_2_/CoS/NC at 5 A g^−1^.


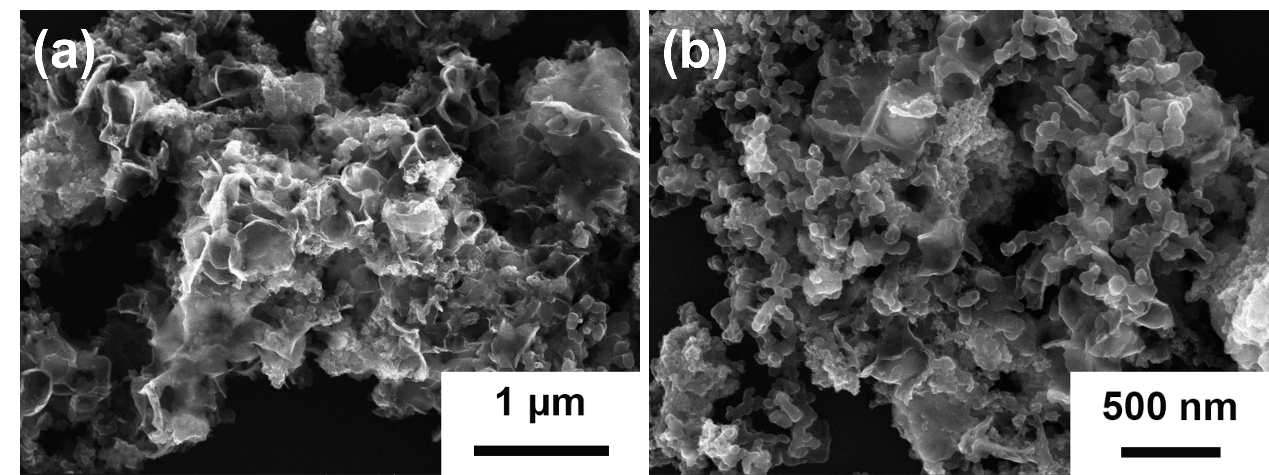


**Figure S11** (a-b) the postmortem SEM images of HC@CoS_2_/CoS/NC after 700 cycles at 15 A g^−1^.


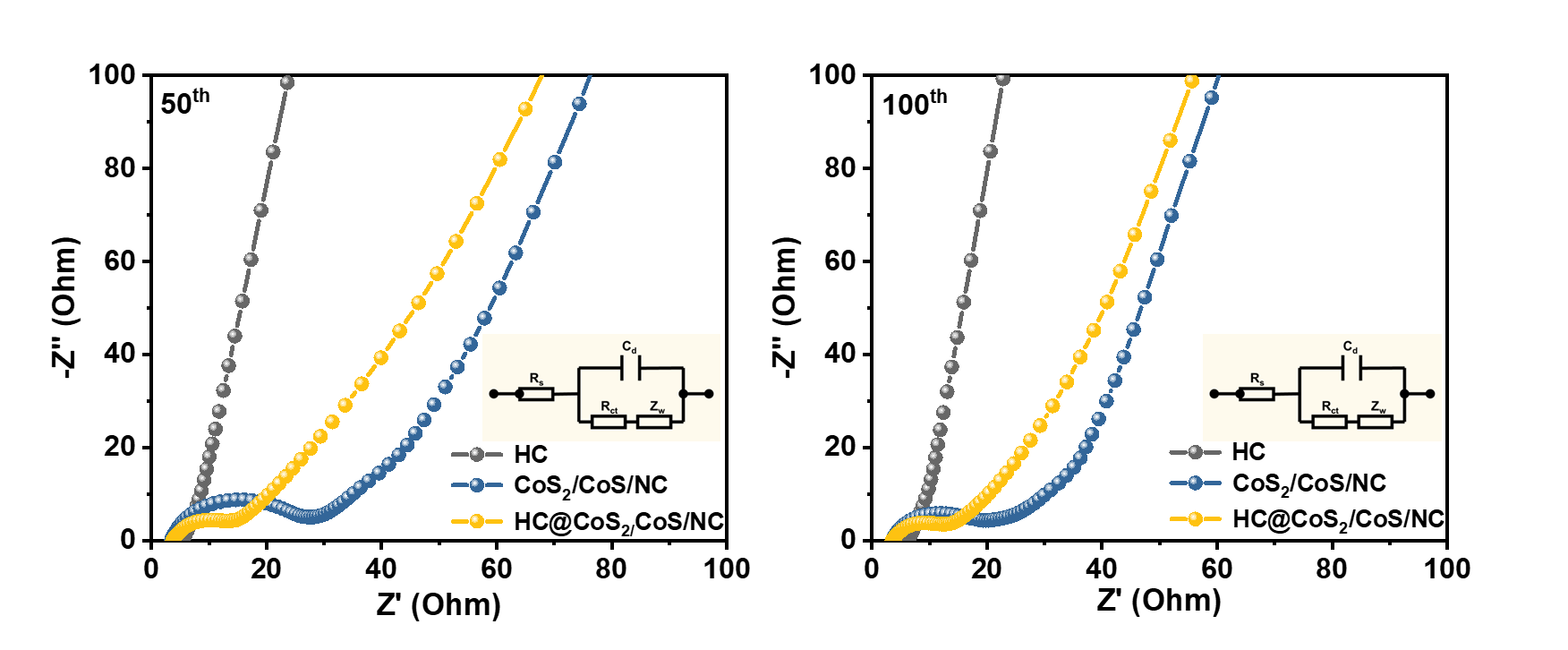


**Figure S12.** Nyquist plots of three samples of a) 50th cycle and b) 100th cycle.

**
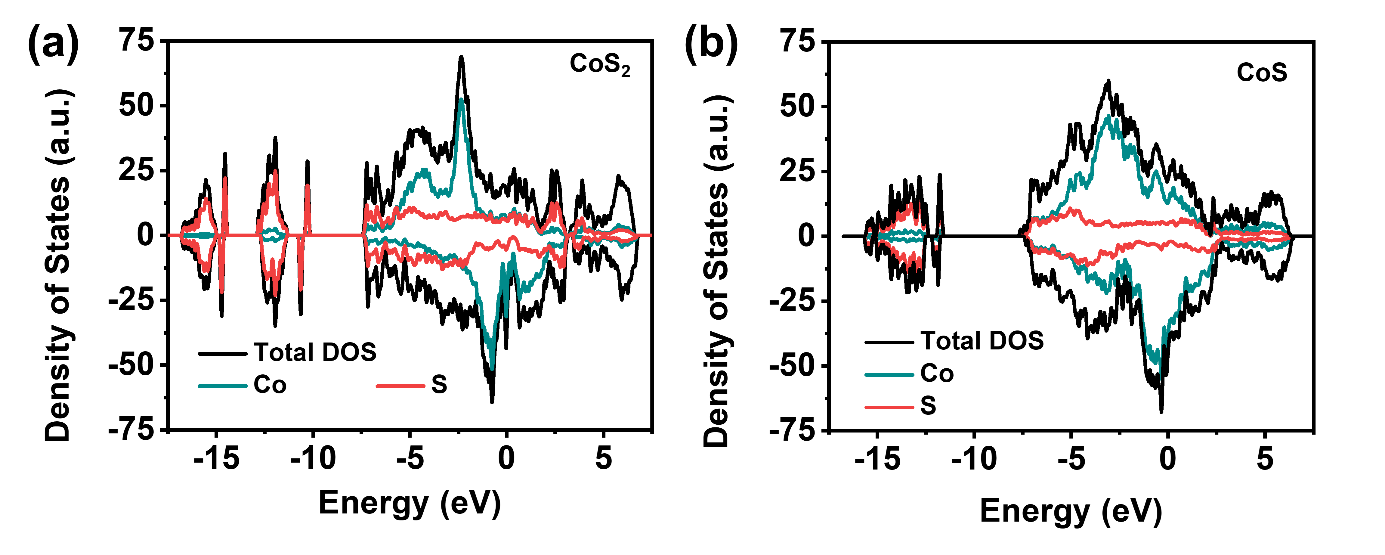
**

**Figure S13** Projected density of states for (a) CoS_2_ and (b) CoS.


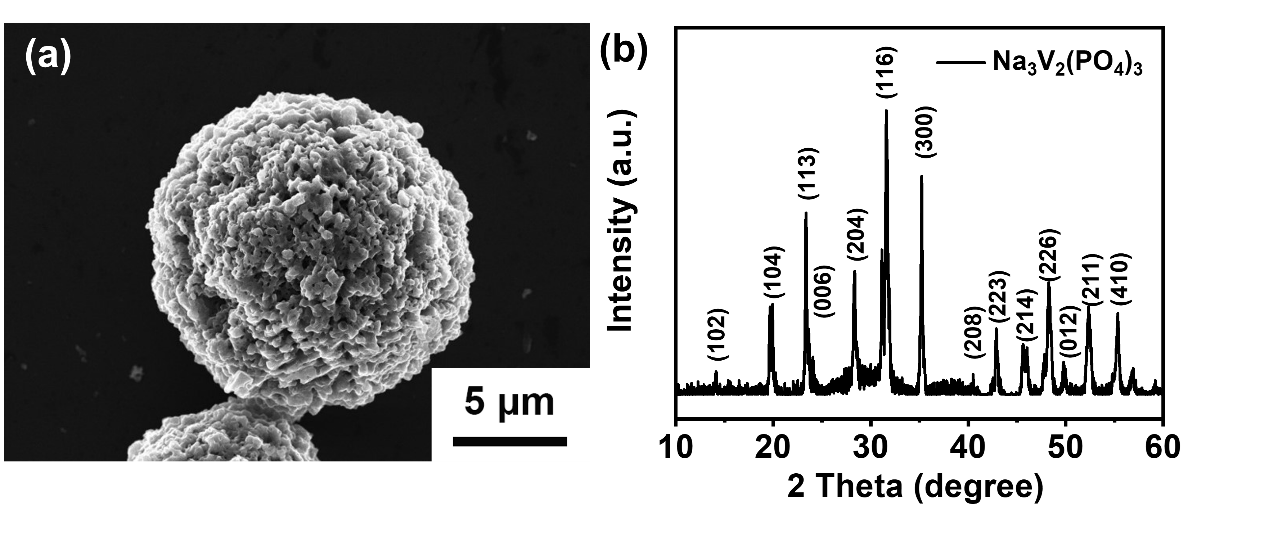


**Figure S14** (a) The SEM image and (b) XRD pattern of Na_3_V_2_(PO_4_)_3_.


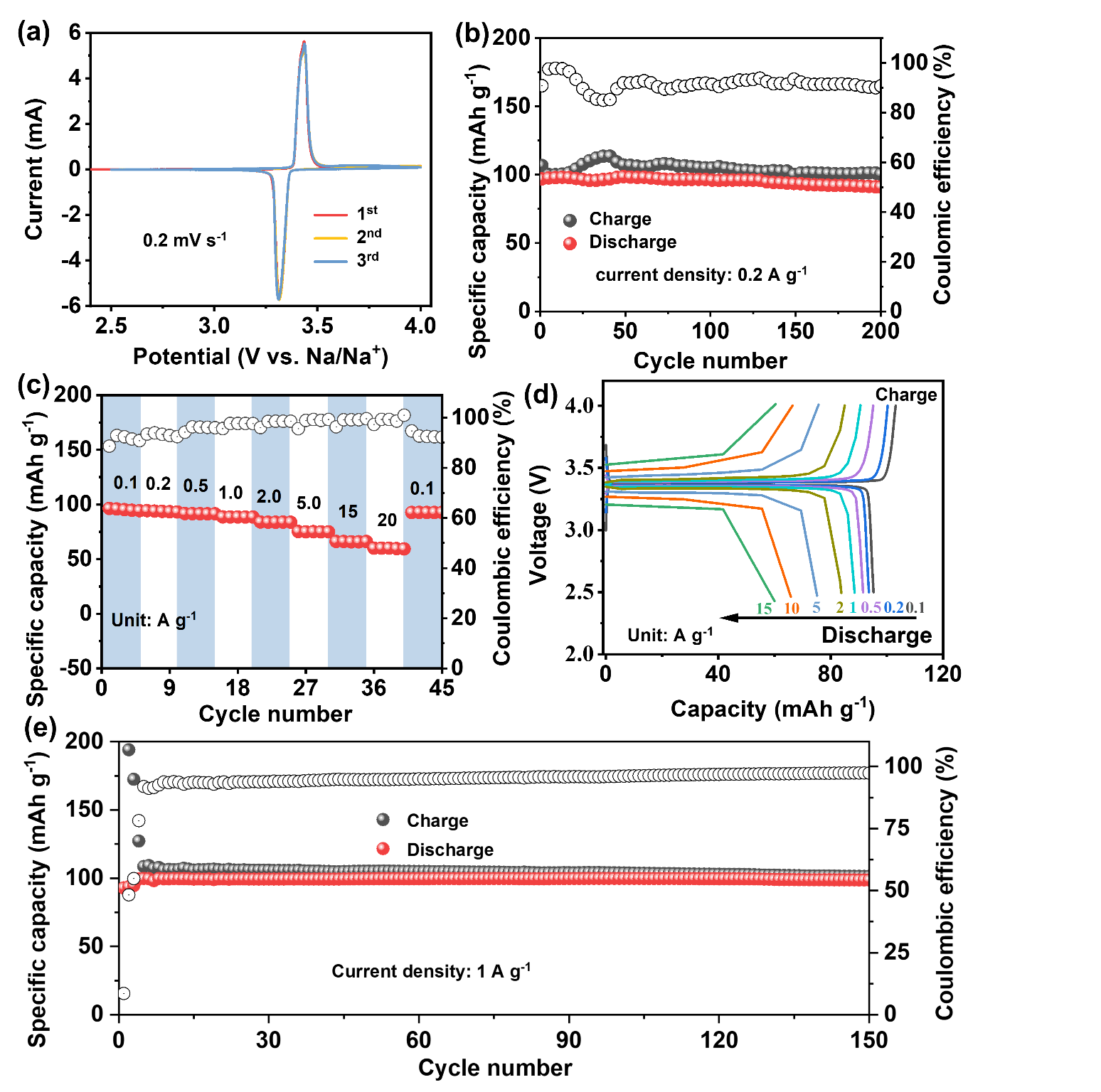


**Figure S15** NVP cathodes: (a) CV curves, (b) cycling performance at 0.1 A g^-1^, (c) rate capabilities, (d) the corresponding representative discharge-charge curves, and (e) cycling performance at1 A g^-1^.

**Table S2** The cycling performances comparisons of HC@CoS_2_/CoS/NC with previously reported Co-based chalcogenide anode for SIBs.

| **Material** | **Voltage range (V)** | **Current density**  **(A g^-1^)** | **Cycle number** | **Specific Capacity**  **(mAh g^-1^)** | **Reference** |
| --- | --- | --- | --- | --- | --- |
| HC@CoS_2_/CoS/NC | 0.01-3.0 | 0.2 | 500 | 716.9 | This work |
|  |  | 2 | 500 | 647.8 |  |
|  |  | 5 | 300 | 637.9 |  |
|  |  | 10 | 700 | 531.0 |  |
| CoS_2_@C nanocubes | 0.01-3.0 | 5 | 200 | 367.6 | Ref [1] |
| CoS_2_-C/CNT | 0.01-3.0 | 0.1 | 200 | 403.0 | Ref [2] |
| CoS_2_@NCNTs | 0.01-2.5 | 0.5 | 200 | 621.5 | Ref [3] |
| CoS_2_/NiS_2_-RGO | 0.01-3.0 | 0.05 | 100 | 473.7 | Ref [4] |
| CuS@CoS_2_ DSNBs | 0.4-2.5 | 0.5 | 500 | 381.57 | Ref [5] |
| CoS_2_–MWCNT | 1.0-2.9 | 0.1 | 100 | 568.0 | Ref [6] |
| CoS_2_/NC@VS_4_ | 0.3-3.0 | 1 | 700 | 307.0 | Ref [7] |
| CoS_2_/NC@NPG | 0.1-3.0 | 1 | 300 | 462.0 | Ref [8] |

**Table S3** The rate performances comparisons of the HC@CoS_2_/CoS/NC with reported CoS_x_-based anodes for SIBs.

| **Material** | **Current density**  **(A g^-1^)** | **Specific Capacity**  **(mAh g^-1^)** | **Reference** |
| --- | --- | --- | --- |
| HC@CoS_2_/CoS/NC | 0.1/0.2/0.5/1/2/  5/10/15/20 | 833.5/728.2/694.4/664.0/638.5/  596.2/539.5/481.7/430.5 | This work |
| CoS_2_@C nanocubes | 0.1/0.2/0.4/0.6  /0.8/1/2/5/10 | 644.2/612.5/576.6/557.6/  528.8/514.3/472.2/412.2/302.8 | Ref [1] |
| CoS@BHCS | 0.5/1/2/3/4/ 5 | 652/594/512/441/389/334 | Ref [9] |
| CoS_2_-C/CNT | 0.05/0.1/0.2/0.5/ 1/1.5/2 | 484/458/417/372  /342/322/306 | Ref [2] |
| Spongy CoS_2_/C | 0.05/0.1/0.2/0.5/1.0/1.5/2.0/4.0 | 563.2/510.4/480.7/370.6/  260.8/184.3/120.7/ 80.9 | Ref [10] |
| CoS/MXene | 0.1/0.2/0.5/1/2/5 | 508/454/405/365/323 | Ref [11] |
| AGC-CoS_2_@NCNFs | 0.1/0.2/0.4/0.8  /1.6/3.2 | 425/379/327/278  /241/201 | Ref [12] |
| P-CoS | 0.2/0.5/1/2/3  /4/5/10 | 572/503/503/483/476  /470/454/389 | Ref [13] |
| CoS_2_/BNG | 0.2/0.5/1/2/5/10 | 520/484/45/439/413/387 | Ref [14] |
| NC@CoS_2_@CNTs | 0.1/0.2/0.5/1/2  /3/5 | 750/669/578/508/437  /378/334 | Ref [15] |

**Supplementary References:**

[1] Z. Zhao, S. Li, C. Li, Z. Liu, D. Li, *Appl. Surf. Sci.* **2020,** *519*, 146268.

[2] Y. Ma, Y. Ma, D. Bresser, Y. Ji, D. Geiger, U. Kaiser, C. Streb, A. Varzi, S. Passerini, *ACS Nano* **2018,** *12* (7), 7220-7231.

[3] Z. Zhang, Y. Huang, X. Gao, Z. Xu, X. Wang, *ACS Appl. Energy Mater.* **2020,** *3* (7), 6205-6214.

[4] J. Liu, Y.-G. Xu, L.-B. Kong, *J. Mater. Sci. Mater. Electron.* **2020,** *31* (12), 9946-9959.

[5] Y. Fang, B. Y. Guan, D. Luan, X. W. Lou, *Angew. Chem. Int. Ed.* **2019,** *58* (23), 7739-7743.

[6] Z. Shadike, M.-H. Cao, F. Ding, L. Sang, Z.-W. Fu, *ChemComm* **2015,** *51* (52), 10486-10489.

[7] X. Li, H. Liang, B. Qin, M. Wang, Y. Zhang, H. Fan, *J. Colloid Interface Sci.* **2022,** *625*, 41-49.

[8] X. Xu, L. Xu, P. Zhang, J.-J. Zhou, W. Wang, W. Wang, Y. Yang, H. Ding, W. Ji, L. Chen, *J. Electroanal. Chem.* **2022,** *921*, 116657.

[9] S. Chen, J. Zhao, Y. Pang, S. Ding, *Nanotechnology* **2019,** *30* (42), 425402.

[10] Y. Zhang, N. Wang, C. Sun, Z. Lu, P. Xue, B. Tang, Z. Bai, S. Dou, *Chem. Eng. J.* **2018,** *332*, 370-376.

[11] Y. Zhang, R. Zhan, Q. Xu, H. Liu, M. Tao, Y. Luo, S. Bao, C. Li, M. Xu, *Chem. Eng. J.* **2019,** *357*, 220-225.

[12] W. Zhang, Z. Yue, Q. Wang, X. Zeng, C. Fu, Q. Li, X. Li, L. Fang, L. Li, *Chem. Eng. J.* **2020,** *380*, 122548.

[13] B. Wang, Y. Cheng, H. Su, M. Cheng, Y. Li, H. Geng, Z. Dai, *ChemSusChem* **2020,** *13* (16), 4078-4085.

[14] X. Cheng, D. Li, F. Liu, R. Xu, Y. Yu, *Small Methods* **2019,** *3* (4), 1800170.

[15] Y. Li, R. Guo, Y. Sun, Y. Wang, W. Liu, H. Pei, H. Zhao, J. Zhang, D. Ye, J. Xie, J. Kong, *ChemElectroChem* **2020,** *7* (13), 2752-2761.
